# TENS alone or combined with low-level laser therapy photobiomodulation for pain, functional capacity, and cardiorespiratory physiological variables after cesarean section: protocol for a randomized clinical trial

**DOI:** 10.1371/journal.pone.0325254

**Published:** 2025-06-20

**Authors:** Alane Macatrão Pires de Holanda Araújo Sena, Ivan Daniel Bezerra Nogueira, Kássio Rafael Rocha de Sena, Patrícia Angélica de Miranda Silva Nogueira

**Affiliations:** 1 Physical Therapy Postgraduate Program (PPGFIS), Federal University of Rio Grande do Norte (UFRN), Natal, Rio Grande do Norte, Brazil; 2 Department of Physical Therapy, Federal University of Rio Grande do Norte (UFRN), Natal, Rio Grande do Norte, Brazil; 3 Physiotherapist, Postgraduate studies, Estácio College in Natal, Rio Grande do Norte, Brazil; Massachusetts General Hospital, UNITED STATES OF AMERICA

## Abstract

**Introduction:**

Women undergoing cesarean section frequently report postpartum pain from the surgical procedure, which may be related to functional mobility and cardiorespiratory physiological variables. Transcutaneous electrical nerve stimulation (TENS) and low-level laser therapy (LLLT) photobiomodulation are electrophysical agents used for analgesic purposes in post-cesarean. However, the combined effect of these interventions and their impact on functional performance and cardiorespiratory physiological variables in this population is not known. This study aims to evaluate the effect of TENS combined with LLLT photobiomodulation on pain, functional performance, and behavior of cardiorespiratory variables and compare it with the effects of TENS alone in women during postpartum after cesarean section.

**Methods:**

This is a protocol of a randomized clinical trial that will be conducted at Divino Amor Maternity Hospital (Rio Grande do Norte, Brazil). A total of 88 patients undergoing cesarean section will be distributed in four groups: TENS combined with LLLT photobiomodulation, TENS alone, placebo, and control. The patients included in the study will be evaluated at three time-points: assessment 1 (8–12 hours postpartum), assessment 2 (20–24 hours postpartum), and assessment 3 (44–48 hours postpartum). Data will encompass sociodemographic and clinical information, pain assessment, functional performance, and cardiorespiratory physiological variables (systemic arterial pressure, heart and respiratory rates, and oxygen saturation). The combined intervention group will follow the protocol of two sessions with low-intensity TENS (30 min, 100 Hz, and 75 µs), with electrodes above and below the cesarean incision, and LLLT photobiomodulation (660 nm, 2 J per point, and 100 mW), without touching the cesarean incision. The TENS alone group will follow the same low-intensity TENS protocol used in the combined intervention. Interventions (combined and isolated) will be delivered after completion of assessments 1 and 2.

**Brazilian Registry of Clinical Trials number:**

RBR-9pjd2n3.

## Introduction

The post-cesarean section period is characterized by the presence of complaints such as pain in the abdominal region and limited movements, especially in the first 24 hours after birth [1–3]. Pain in the cesarean incision region is considered post-cesarean morbidity for the mother and infant, characterized as postsurgical pain that may be challenging to recover from. This pain is related to the inflammatory phase of the tissue repair process, which can lead to difficulties in positioning the infant for breastfeeding, caring for the newborn, and performing activities such as sitting, standing, walking, and intimate hygiene [4,5].

Scientific studies identify therapeutic resources as interventions for analgesia, evaluating the possible correlation between pain scores and functional capacity and cardiorespiratory physiological variables (blood pressure, heart and respiratory rates, and oxygen saturation) post-intervention [6,7]. In the literature, transcutaneous electrical nerve stimulation (TENS) [1,8] and photobiomodulation with low-level laser therapy (LLLT) [9,10] are non-pharmacological resources that promote analgesia in the immediate postpartum period after cesarean section.

TENS is a non-invasive, safe, and low-cost treatment modality that uses electrical currents of different frequencies distributed through electrodes placed on the skin surface. The effectiveness of TENS analgesia relates to the gate theory and the activation of the endogenous opioid system [1,11]. In turn, LLLT photobiomodulation utilizes photons in non-thermal irradiance to stimulate biological activity, increasing the production of endogenous opioid neurotransmitters, adenosine triphosphate, anti-inflammatory cytokines, and local angiogenesis, in addition to improving thermal pain. This therapy activates numerous intracellular signaling pathways and alters the affinity of transcription factors related to cell proliferation, survival, tissue repair, and regeneration [9].

In another clinical trial, 90 women post-cesarean were distributed in two groups (TENS and control). For the TENS group, high-frequency, low-intensity TENS (30 min, 100 Hz, 75 μs, sensory threshold, and segmental application) was applied in the immediate post-cesarean period, distributed in two sessions with a 24-hour interval between them, and electrodes placed above and below the cesarean incision. The results showed reduced complaints of pain in the abdominal region associated with the incision and postpartum uterine contractions [1]. Thus, TENS is recommended to improve pain in patients undergoing cesarean section, allowing better execution of activities that require movement in the acute postsurgical phase [1,11,12]. Furthermore, in addition to contributing to functional capacity after cesarean section, a Cochrane review observed that TENS may also reduce heart and respiratory rates [7].

A study verified the analgesic effects of LLLT photobiomodulation in 88 women after cesarean section, randomly distributed in four groups (intervention with 2 J, and intervention with 4 J, placebo, and control). Two sessions of LLLT photobiomodulation (660 nm) were applied, the first at 8–12 hours and the second at 20–24 hours postpartum. The patients reported reduced pain in the cesarean incision after intervention and a perception of global improvement using the Perception of Global Change Scale. According to the authors, the LLLT photobiomodulation with 2 J presented a larger effect size than the other groups [9].

TENS and LLLT photobiomodulation are non-invasive resources and alone can promote analgesia when applied in the first post-cesarean days. Furthermore, they can modify functional mobility and cardiorespiratory physiological variables, such as blood pressure, heart and respiratory rates, and oxygen saturation. However, no studies were found in the literature that verified the effects of combined therapy of these resources in post-cesarean women and how this combined therapy could act on cardiorespiratory physiological variables and functional performance in the target audience of future research. It is important that the physiotherapist performs a procedure that addresses the main complaints, considering the patient’s clinical condition. Thus, the hypothesis of this study is that therapy with TENS combined with LLLT photobiomodulation promotes improvement in functional performance and alters the behavior of cardiorespiratory physiological variables through reduced pain in the cesarean incision in the first post-cesarean days and thus strengthen evidence-based physiotherapy, using scientifically proven physiotherapeutic resources in clinical practice, stimulating the realization of new work related to the subject. The assessed outcomes will encompass pain, functional capacity, and the behavior of cardiorespiratory physiological variables after the intervention.

### Objectives

#### Primary objective.

This study aims to analyze the effect of TENS combined with LLLT photobiomodulation on pain outcomes in women after cesarean section.

#### Secondary objective.

This study aims to evaluate the influence of TENS combined with LLLT photobiomodulation on the outcomes functional capacity, systemic arterial pressure, respiratory and heart rates, oxygen saturation.

## Materials and methods

This randomized controlled clinical trial will be conducted at Divino Amor Maternity Hospital (Rio Grande do Norte, Brazil), and patients will be recruited according to the convenience of admission for cesarean birth and distributed into one of the 4 study groups (TENS alone, TENS combined photobiomodulation LLLT, Placebo or Control). This Study, the recruitment started in the July 2023 and not has been completed, the estimate to data collection be completed is august 2025, and results are expected decision-makers in resource allocation for improved care for this population, aiming to obtain insights into the most effective recuperation in postpartum.

To sample calculation, the study sample was the result of a probabilistic sampling process. For each group, the sample number was 17 volunteers, adding 20% of sample losses, the final number for each group was 22 volunteers. Thus, the sample size was 88 participants, based on the degree of pain in the surgical incision region (according to the numeric pain scale [NPS], graded from 0 to 10, and a reduction of 2 points in the pain score) and variable standard deviation for each group of 1.5. The alpha error was 0.05, the test power was 80% [9,13].

Eligibility criteria will encompass women within 8–12 hours post-cesarean birth; only woman habitual risk pregnancy; presence of pain NPS ≥ 3 in the cesarean incision region; no clinical or obstetric complications; no infectious process; undergoing drug treatment (anti-inflammatories, analgesics, and anti-gas drugs) with the same intake interval; absence of neurological or musculoskeletal disorders or alterations; and not physically active.

Exclusion criteria will encompass discontinuation of TENS or the LLLT photobiomodulation interventions; failure to perform the 2-min walk test (2MWT) during the assessment period; presence of clinical or obstetric instability during the assessment period; or patients who have already undergone TENS intervention or have a cognitive deficit.

The sample will be randomized (www.randomization.com) in four groups: TENS intervention combined with LLLT photobiomodulation, TENS alone, placebo, and control. The groups will be coded, and allocation will be done in sealed and opaque envelopes, numbered consecutively.

This study will have three investigators: the first will be responsible for randomization, the second for assessments, and the third for delivering the intervention. This will be a blinded study because the second investigator and the patients will be unaware of the allocation and intervention.

**Fig 1:**
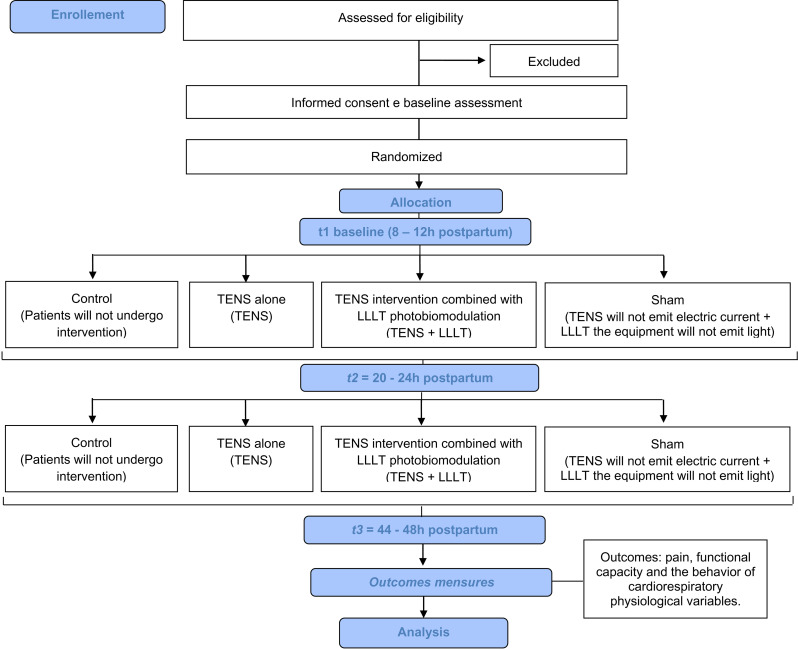
Flow chart of study protocol. Flow chart of study protocol. CONSORT 2010 FLOW DIAGRAM.

This protocol was approved by the research ethics committee of the Federal University of Rio Grande do Norte (Brazil) (CAAE: 53263721.5.0000.5537) and submitted to the Brazilian Registry of Clinical Trials (RBR-9pjd2n3). The autonomy and anonymity of patients will be respected, and personal data privacy will be ensured following resolution no. 510/16 of the National Health Council and the Declaration of Helsinki. Before entering the study, all patients will sign the informed consent form for written. The flow chart of study protocol is described in Fig 1 (Fig 1).

Our study followed the standard protocol items: recommendations for interventional trials (SPIRIT), which aims to improve clinical trial quality (S1 Checklist, SPIRIT 2013 Checklist) *.

The patients included will be evaluated at three time points: t1 (8–12 h post-cesarean) will use an evaluation form, International Physical Activity Questionnaire (IPAQ) application, pain intensity, pressure pain tolerance and threshold, functional capacity, and cardiorespiratory physiological variables evaluation; t2 (20–24 h post-cesarean) and t3 (44–48 h post-cesarean) will evaluate pain intensity, pressure pain tolerance and threshold, functional capacity, and cardiorespiratory physiological variables.

After t1 and t2, patients allocated to intervention groups will receive their respective protocol.

### Data collection

**Fig 2:**
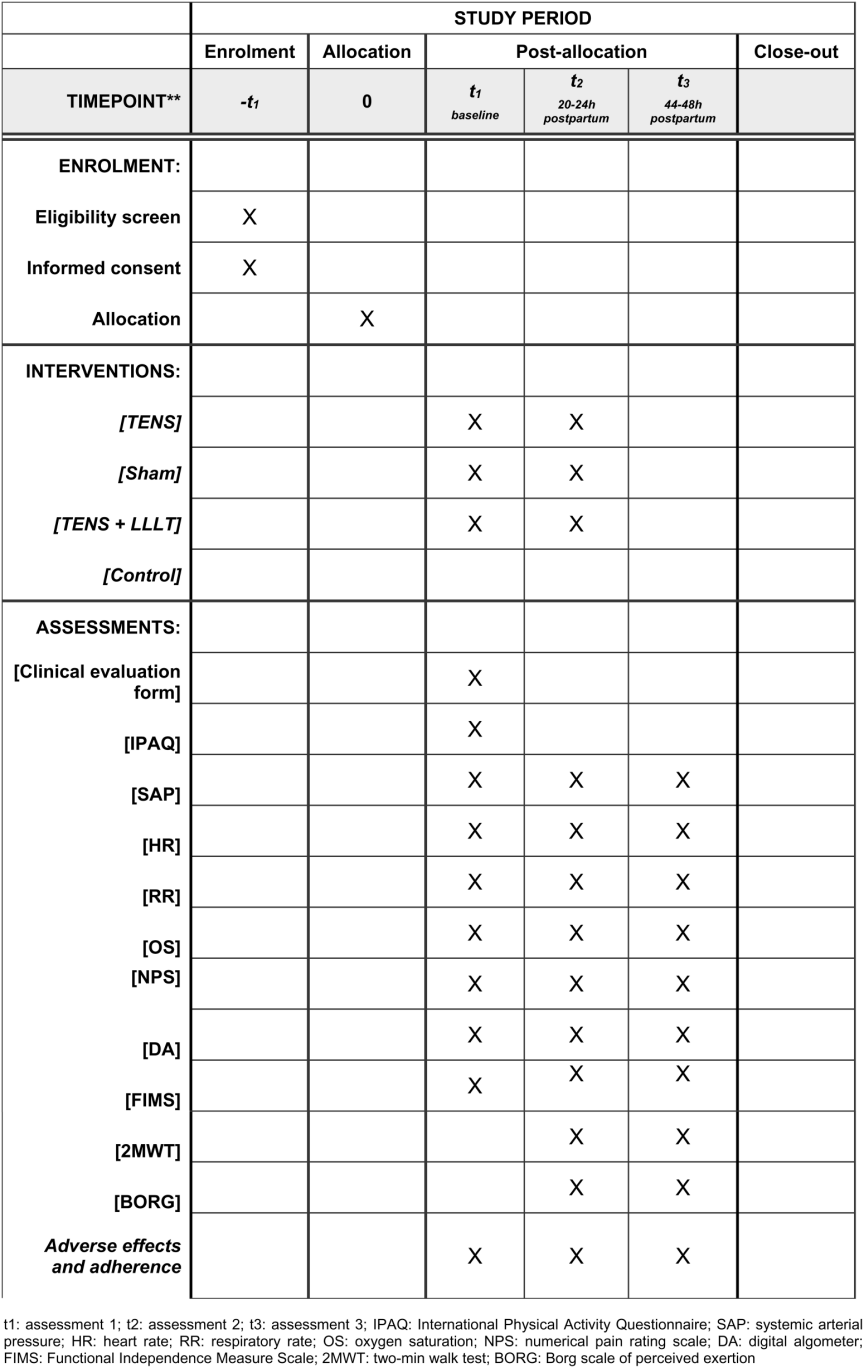
Template of recommendations of enrollment, interventions, and assessments schedule. t1: assessment 1; t2: assessment 2; t3: assessment 3; IPAQ: International Physical Activity Questionnaire; SAP: systemic arterial pressure; HR: heart rate; RR: respiratory rate; OS: oxygen saturation; NPS: numerical pain rating scale; DA: digital algometer; FIMS: Functional Independence Measure Scale; 2MWT: two-min walk test; BORG: Borg scale of perceived exertion.

The Fig 2 details the assessments and outcome variables.

### Clinical evaluation form

The patients will respond to an evaluation form. This form was designed for specific use in this study and considers sociodemographic data (name, address, age, educational level, marital status, associated diseases, and history of coronavirus infection); obstetric data (gestational age, parity, labor, date of birth, and postpartum period); clinical data (level of physical activity, pain intensity, pressure pain tolerance and threshold, functional independence, walking distance covered in the 2MWT, and subjective perception of effort).

### International physical activity questionnaire (IPAQ)

The IPAQ will be used to assess the level of physical activity. This questionnaire was validated in Brazil and classifies the patients as very active, active, irregularly active, or sedentary. Those who do not perform physical activity for at least 10 min during the week will be considered sedentary [14].

### Cesarean incision pain

In assessments 1, 2, and 3, the NPS and digital algometer will assess pain in the cesarean incision region.

Pain intensity will be assessed using the NPS [9,15]. This scale allows the patient to classify pain from level 0 (no pain) to level 10 (extreme pain). The evaluation of pain will follow verbal descriptors (0 = no pain; 1–3 = mild pain; 4–6 = moderate pain; and 7–10 = extreme pain). Pain in the abdominal region will be assessed with the patient at rest and in movement (one minute after the 2MWT).

Pressure pain tolerance and threshold will be assessed by a digital force gage algometer (WAGNER FDM model), a device with a 1 cm^2^ rubber disc connected to a pressure gauge, which displays values in kgf/cm^2^ [9]. The patient will be instructed to say “started” when the pressure applied by the device begins to evoke pain (threshold), and the pressure will continue until the patient says “stop” (tolerance). The pressure will be applied perpendicularly at the midpoint between the navel and the cesarian incision, taking the linea alba as a reference. The rubber disc will be covered with plastic film and cleaned with 70% alcohol before each patient.

### Functional independence measure (FIM)

This study will evaluate the functional independence of the patient in the TENS combined with LLLT photobiomodulation group. The FIM is an instrument validated for the Portuguese language and classifies patients according to their ability to perform an independent activity, quantifying their need for assistance [15].

### Functional capacity (2MWT)

The 2MWT will assess the distance (meters) covered during 2 min of walking and will be used for measuring functional capacity [16]. The patient will be asked to walk for 2 min in a 30-m corridor, with the time recorded with a digital stopwatch and distances delimited with tape on the floor. Patients will be encouraged to walk as fast as possible without running, respecting their limits. The examiner will stay half a meter behind the patient to ensure safety. Two practical attempts will be performed, and the 2MWT with the longest distance will be recorded in the assessment. A 30-minute break between the two attempts will be given for the patients to rest [16,17]. Cardiorespiratory variables will be monitored before and immediately after the 2MWT, as well as the self-reported perception of effort.

### Subjective perception of effort

Patients will be asked about their subjective perception of respiratory effort before and during the 2MWT using the Borg scale of perceived exertion [18]. This scale is graded from 6 (no exertion at all) to 20 (maximal exertion). Higher values correspond to a greater sensation of dyspnea.

### Blood pressure, heart and respiratory rates, and oxygen saturation

Physiological cardiorespiratory variables, such as blood pressure, heart and respiratory rates, and oxygen saturation, will be evaluated at t1. In t2 and t3, this evaluation will be performed before, during, and after the 2MWT.

### Intervention protocols

**Fig 3:**
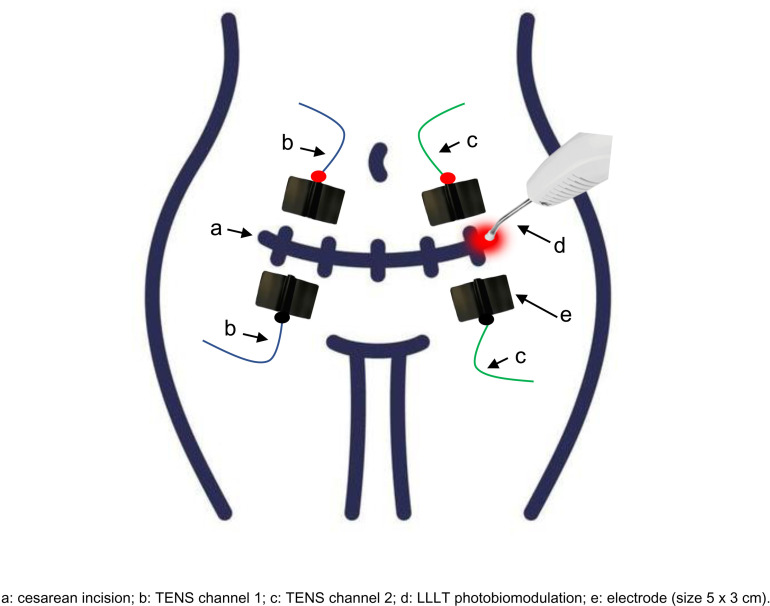
TENS combined with LLLT photobiomodulation. a: cesarean incision; b: TENS channel 1; c: TENS channel 2; d: LLLT photobiomodulation; e: electrode (size 5 x 3 cm).

Protocol for TENS combined with LLLT photobiomodulation: Both interventions will be applied simultaneously, with the patient positioned in the supine position and a neutral posture. We will follow a TENS intervention protocol previously described [1], consisting of low-intensity TENS (30 min, 100 Hz, and 75 µs) applied using two channels and four electrodes (size 5 x 3 cm) placed in the skin surface with conductive gel above and below the cesarean incision, sanitized with 70% alcohol. The LLLT photobiomodulation intervention protocol will follow a reference from the literature [9], consisting of 660 nm (red), energy of 2 J per point, and power of 100 mW, performed in two sessions without skin contact in the cesarean incision. The LLLT intervention will be punctual and applied perpendicularly to the skin surface in the line of the cesarean incision [8]. The number of points applied will depend on the extent of the cesarean incision. The time per point will be 20 seconds, and the distance of 1 cm between application points will be respected. To perform the application with LLLT photobiomodulation, the application pen will be cleaned with 70% alcohol before each patient. Asepsis in the cesarean incision region will also be performed using 70% alcohol before and after the intervention. Only the therapist and the patient will be present during this application due to the need for specific protective glasses for the LLLT. The Fig 3 represent the procedure to application TENS combined with LLLT photobiomodulation.

Protocol for TENS alone: patients will receive the same TENS intervention parameters and positioning previously described in the TENS combined with LLLT photobiomodulation protocol.

Protocol for the placebo: Patients will follow the same TENS combined with LLLT photobiomodulation protocol as in the intervention group, but the equipment will not emit light or electric current.

Protocol for the control: Patients will only undergo t1, t2, and t3.

### Statistical analysis

Data analysis will be performed using SPSS software version 22.0 (IBM Corp. USA), and in all statistical analyses, p values < 0.05 were considered as statistically significant. Will be performed Shapiro-Wilk test and Levene for normality of the data distribution and equality of variances. Descriptive statistics will be presented using the measures of central tendency. Mauchly’s test of sphericity will be used to validate the correlation of the repeated measures, and if the assumption of sphericity was violated, the Greenhouse-Geisser correction will be applied. Two-way repeated measures ANOVA will be to compare the effects of TENS combined with LLLT photobiomodulation between groups (TENS alone, Placebo and Control) over time (assessment t1, t2 and t3) on a primary outcome (pain) and secondary outcomes (functional capacity, systemic arterial pressure, respiratory and heart rates, oxygen saturation and Functional Independence Measure). When appropriate, post hoc comparisons will be carried out using Tukey’s post hoc correction for multiple comparisons. The effect size will be calculated by Cohen’s. For the missing data, intention to treat analysis will be used.

For better data management and quality control the investigator responsible for randomization will be responsible for organization the collected patients information in spreadsheets and ensure confidentiality without personal identification of the research participants.

## Supporting information

S1 Checklist SPIRIT 2013 Checklist: recommended items to address in a clinical trial protocol and related documents*. (DOC)

S1 File Research project submitted to the ethics committee. (ZIP)
