# Supplementary material for: TENS alone or combined with low-level laser therapy photobiomodulation for pain, functional capacity, and cardiorespiratory physiological variables after cesarean section: protocol for a randomized clinical trial
Source: PLoS One. 2025 Jun 20;20(6):e0325254. doi: 10.1371/journal.pone.0325254 (PMC12180720; doi:10.1371/journal.pone.0325254)
Supplement: S1 File — (ZIP) [file pone.0325254.s002.zip › Study complete_approved in version original.pdf]

UNIVERSIDADE FEDERAL DO RIO GRANDE DO NORTE
CENTRO DE CIÊNCIAS DA SAÚDE
PROGRAMA DE PÓS-GRADUAÇÃO EM FISIOTERAPIA

INFLUÊNCIA NO DESEMPENHO FUNCIONAL DA TERAPIA COMBINADA DE LASER E TENS NA INCISÃO CESARIANA COM QUADRO ÁLGICO: ensaio clínico randomizado

Alane Macatrão Pires de Holanda Araújo Sena

NATAL - RN
2021

Alane Macatrão Pires de Holanda Araújo Sena

INFLUÊNCIA NO DESEMPENHO FUNCIONAL DA TERAPIA COMBINADA DE LASER E TENS NA INCISÃO CESARIANA COM QUADRO ÁLGICO: **ensaio clínico** randomizado

Projeto apresentado ao Programa de Pós-graduação em Fisioterapia da Universidade Federal do Rio Grande do Norte para seleção no Doutorado em Fisioterapia.

Linha de Pesquisa: Avaliação e intervenção em Fisioterapia nos sistemas cardiovascular e respiratório.
Orientadora: Profª. Dra. Patrícia Angélica de Miranda Silva Nogueira

NATAL - RN

2021

SUMÁRIO

RESUMO 13

ABSTRACT 14

1. INTRODUÇÃO 9

2. JUSTIFICATIVA 11

3. OBJETIVOS 12

3.1 Objetivo geral 12

3.2 Objetivos específicos 12

4. HIPÓTESES 13

5. **MATERIAIS** E MÉTODOS 14

5.1 Caracterização da pesquisa 14

5.2 Procedimentos Éticos 14

5.3 População e amostra 14

5.3.1 Cálculo amostral 15

5.3.2 Critérios elegibilidade 15

5.3.2.1 Critérios de Inclusão 15

5.3.1.2 Critérios de Exclusão 16

5.4 Instrumentação 16

5.5 Procedimentos para a coleta 20

5.5.1 Dias e horários das coletas 23

5.6 Análise estatística 23

5.7 Fluxograma do estudo 24

6. CRONOGRAMA 25

7. RESULTADOS ESPERADOS E PERSPECTIVAS PARA O AVANÇO DO CONHECIMENTO 26

8. RISCOS E BENEFÍCIOS 27

9. ORÇAMENTO 28

10. REFERÊNCIAS 29

APÊNDICE 32

APÊNDICE A - TERMO DE CONSENTIMENTO LIVRE E ESCLARECIDO (TCLE) 32

APÊNDICE B - FICHA DE AVALIAÇÃO 37

ANEXO 38

ANEXO A - ESCALA NUMÉRICA DA DOR 38

ANEXO B - ESCALA DE PERCEPÇÃO GLOBAL DE MUDANÇA (PGIC VERSÃO PORTUGUESA) 39

ANEXO C - QUESTIONÁRIO INTERNACIONAL DE ATIVIDADE F)SICA (IPAQ VERSÃO CURTA) 40

RESUMO

Introdução: No puerpério imediato de mulheres submetidas à cesárea é relatada como queixa principal a dor na região abdominal decorrente do procedimento cirúrgico pós o parto gerando desconforto e impacto nas atividades necessárias da puérpera com o bebê e movimentos no próprio leito. Para melhor qualidade de vida das puérperas que se encontram nos primeiros dias de pós parto, é importante que o profissional fisioterapeuta realize uma conduta atuando nas principais queixas considerando a condição clínica da paciente. O LBI e a TENS são recursos não invasivos que estão sendo utilizados nas pesquisas científicas em estudos objetivando analgesia favorecendo aplicabilidade nas práticas clínicas de forma isolada em puérperas, porém não foi encontrado na literatura estudos que verificassem os efeitos da terapia combinada desses recursos em mulheres pós cesárea e sua influência no desempenho funcional. **Objetivo:** Avaliar a influência no desempenho funcional da estimulação elétrica nervosa transcutânea combinada com a terapia laser de baixa intensidade em mulheres submetidas à cesárea com quadro álgico. **Métodos:** A pesquisa será caracterizada como ensaio clínico randomizado e cego. A amostra será composta por mulheres no puerpério imediato pós cesárea, seguindo os critérios de elegibilidade proposto no estudo e distribuídos de forma aleatória em quatro grupos: controle (GC), controle por placebo (GCP), grupo com aplicação de TENS combinado com laser de baixa intensidade (GTL) e grupo com aplicação de TENS (GT). Todos os grupos serão submetidos a três avaliações (dados do prontuário, Escala de Percepção Global de Melhora, Escala Numérica da Dor, o Algômetro Digital, Teste de Caminhada de 2 minutos e Medida de Independência Funcional). O estudo seguirá o protocolo com Laser de Baixa Intensidade de 660nm sem contato na incisão cesariana em duas sessões combinado com a TENS de 100Hz com eletrodos acima e abaixo da ferida operatória. No estudo terá um grupo que será submetido somente com o TENS. Na análise estatística, será feita uma análise descritiva das variáveis do estudo de acordo com o teste de normalidade Kolmogorov-Sminorv. Para comparações de médias entre grupos será utilizado o teste ANOVA e verificadas prováveis diferenças aplicar-se-á o teste de post hoc de Tukey. **Resultados esperados:** É esperado

verificar melhora no desempenho funcional para realizar atividades que exijam movimentos posterior terapia combinada de TENS com Laser de Baixa Intensidade na incisão cesariana, o que permitirá aos fisioterapeutas e comunidade acadêmica utilizar este recurso terapêutico no público alvo desse estudo, assim como favorecerá de evidência cientifica para aplicabilidade na sintomatologia dolorosa em novas pesquisas.

Palavras-chave: Puerpério. Fototerapia. Eletroterapia. Desempenho físico funcional.

ABSTRACT

Introduction: In the immediate puerperium of women undergoing cesarean section, pain in the abdominal region resulting from the postpartum surgical procedure is reported, causing discomfort and impact on the puerperal woman's necessary activities with the baby and movements in the bed itself. For a better quality of life for the mothers who are in the first days of postpartum, it is important that the physiotherapist perform a conduct acting on the main complaints considering the clinical condition of the patient. LBI and TENS are non-invasive resources that are being used in scientific research in studies aimed at analgesia, favoring applicability in clinical practices in isolation in puerperal women, but studies that verified the effects of combined therapy of these resources in postpartum women were not found in the literature. cesarean section and its influence on functional performance. Aim: Evaluate the influence on the functional performance of transcutaneous electrical nerve stimulation combined with Iow-intensity laser therapy in women undergoing cesarean with pain. **Methods:** The research will be characterized as a randomized and blind clinical trial. The sample will consist of women in the immediate post- cesarean postpartum period, following the eligibility criteria proposed in the study and randomly distributed into four groups: control (GC), placebo control (PCG), group with application of TENS combined with laser low intensity (GTL) and group with application of TENS (GT). AII groups will undergo three assessments (data from medical records, Global Perception of Improvement Scale, Numerical Pain Scale, Digital Algometer, 2-minute Walk Test and Functional Independence Measure). The study will follow the protocol with Low Intensity Laser of 660nm without contact in the cesarean incision in two sessions combined with TENS of 100Hz with electrodes

above and below the surgical wound. In the study there will be a group that will be submitted only with TENS. In the statistical analysis, a descriptive analysis of the study variables will be performed according to the Kolmogorov-Sminorv normality test. For comparisons of means between groups will be used the ANOVA test and verified probable differences will apply Tukey's post hoc test. **Expected Results:** It is expected to verify improvement in functional performance to perform activities that require subsequent movements combined with TENS therapy with Low Intensity Laser in the cesarean section, which will allow physiotherapists and the academic community to use this therapeutic resource in the target audience of this study, as well as favoring evidence for applicability in painful symptomatology in new research.

Keywords: Postpartum period. Phototherapy. Eletrotherapy. Physical Functional Performance.

1. INTRODUÇÃO

O pós-parto cesáreo é caracterizado por presença de queixas como dor na região abdominal e limitação de atividades que exijam movimentos principalmente nas primeiras 24 horas pós o parto[1 2 3]. A dor na região da incisão cesariana é considerada como morbidade pós cesárea para mãe e o bebê, caracterizada como dor pós-operatória que apresenta difícil recuperação. A dor na ferida operatória está relacionada com a fase inflamatória do processo de reparo tecidual e dessa forma resulta em dificuldade nos posicionamentos de amamentar, cuidados com o recém-nascido e para atividades como sentar, levantar, caminhar e realizar higiene íntima[4,5]

Um estudo realizado com aplicação do questionário WHODAS-12, utilizado para avaliação do grau de dificuldade para realizar atividades apresentado nos últimos 30 dias pelo indivíduo, foi aplicado em muheres no pós parto, e verificado em seus resultados a presença de dificuldade nas mobilidades (como ficar de pé e durante o andar) com impacto na amamentação[6].

É verificado, em alguns estudos, recursos não farmacológicos que são sugeridos em terapias para quadros clínicos no puérperio imediato (período que compreende o primeiro dia ao décimo dia pós o parto) em mulheres submetidas ao parto cesáreo para favorecer analgesia[4] como Laser de Baixa Intensidade (LBI)’ [8] e Estimulação Elétrica Nervosa Transcutênea (TENS)[1] ^. O LBI consiste em emissão de luz e que promove efeito analgésico em lesões ósseas, musculares e tendíneas agudas ou crônicas, vasodilatação e proliferação de micro vasos, com possível aumento da quantidade de oxigênio no tecido, proliferação epitelial, endotelial e fibroblástica, aumento da síntese de colágeno e da atividade fagocitária, acelerando o processo de reparação tecidual, além da liberação de citocinas que irão reduzir a reação inflamatória[10]. No caso da TENS, este consiste numa corrente elétrica que quando utilizado em alta frequência (50 — 100Hz) promove analgesia e seus efeitos envolvem sistemas de controle da dor através da ativação do sistema opióide endógeno e teoria da comporta (a estimulação de fibras nervosas aferentes de grosso calibre e rápida velocidade de condução são transmitidos para a medula espinhal, onde bloqueiam temporariamente estímulos nociceptivos de dor ao cérebro)[11,12]

Com o intuito de verificar efeitos analgésicos da terapia LBI, foi verificado no estudo realizado com 88 mulheres submetidas à cesareana distribuídas randomicamente em 4 grupos (controle, placebo, intervenção com dose de 2J/cm^2 e intervenção com dose de 4J/cm^2) aplicando duas sessões de LBI com comprimento de onda de 660nm (primeira sessão em 8-12h pós o parto e a segunda sessão em 20-24h pós o parto), redução da dor na ferida operatória pós intervenção com percepção de melhora global referida pela paciente por meio de aplicação da Escala de Percepção Global de Mudança (PGIC versão Portuguesa). Nesse estudo, o LBI com dose de 2J/cm^2 apresentou, em seus resultados, maior tamanho do efeito quando comparado com demais grupos do estudo[7].

No ensaio clínico realizado com 90 voluntárias submetidas a parto cesárea e distribuídas aleatoriamente em dois grupos (grupo controle e grupo submetido a terapia TENS) foi observado que, a terapia TENS colocando os eletrodos abaixo e acima do incisão cesariana utilizando parâmetros com frequência de 100Hz e largura de pulso de 75ps aplicado em duas sessões (30 minutos cada sessão) com intervalo de 24h entre as sessões no pós operatório imediato, promove melhora na queixa de dor relatada pelas puérperas na região abdmonial associada a inscisão cirúrgica e contrações uterinas pós parto[1].

É relatado na literatura que a dor em pós operatório agudo influencia na mobilidade[1], sendo observado que o uso do TENS, com o objetivo de melhorar o quadro álgico em pacientes submetidos à procedimentos cirúrgicos, é susgestivo como terapia para favorecer melhor execução das atividades que exijam movimentos na fase aguda por promover alívio da dor[12].

Diante do quadro álgico com limitação de movimentos nos primeiros dias de pós parto cesárea aliado aos efeitos da TENS encontrados na literatura para analgesia com consequente melhora na execução de movimentos e os achados científicos também acerca da analgesia com o uso da terapia LBI, esse estudo terá o propósito de verificar a possibilidade de aplicação da terapia TENS combinada com LBI na ferida operatória de mulheres submetidas ao parto cesáreo com queixa de dor na melhora do desempenho funcional pós intervenção durante puerpério imediato.

2. JUSTIFICATIVA

No puerpério imediato de mulheres submetidas à cesárea é relatada como queixa principal a dor na região abdominal decorrente do procedimento cirúrgico pós parto gerando desconforto e impacto nas atividades necessárias da puérpera com o bebê e movimentos no próprio leito[4 10].

Para melhor qualidade de vida das puérperas que se encontram nos primeiros dias de pós parto, é importante que o profissional fisioterapeuta realize uma conduta atuando nas principais queixas considerando a condição clínica da paciente. O LBI e a TENS são recursos não invasivos que estão sendo utilizados nas pesquisas científicas em estudos objetivando analgesia favorecendo aplicabilidade nas práticas clínicas de forma isolada em puérperas, porém não foi encontrado na literatura estudos que verificassem os efeitos da terapia combinada desses recursos em mulheres pós cesárea.

Diante do exposto, se faz necessário verificar a possibilidade de utilizar os recursos combinados da terapia LBI e TENS na incisão cesareana para analgesia e sua influência no desempenho funcional das pacientes, para fortalecer a fisioterapia baseada em evidência e estimular a realização de novos trabalhos relacionados ao tema.

3. OBJETIVOS

3.1 Objetivo geral

Avaliar a influência no desempenho funcional da estimulação elétrica nervosa transcutânea combinada com a terapia laser de baixa intensidade em mulheres submetidas à cesárea com quadro álgico.

3.2 Objetivos específicos

- Avaliar a dor na região abdominal em mulheres submetidas à cesariana através da escala numérica da dor ao longo do tempo com a paciente em repouso e em movimento;
- Verificar o limiar de dor na região abdominal em mulheres submetidas à cesariana ao longo do tempo através da algometria;
- Avaliar a melhora da condição clínica através da Escala de Percepção Global de Mudança nas primeiras 24 horas e 48 horas pós o parto;
- Avaliar a independência funcional através da Escala de Medida de Independência Funcional com 8-12h, 20-24h e 44-48h após o parto;
- Avaliar o desempenho funcional através do Teste de 2 (dois) minutos nas primeiras 24 horas e 48 horas após o parto;
- Comparar o desempenho funcional intergrupos ao longo do tempo.

4. HIPÓTESES

HO: A terapia com estimulação elétrica nervosa transcutânea combinada com o laser de baixa intensidade promove melhora no desempenho funcional através do alívio da dor na região abdominal em mulheres submetidas à cesárea no puerpério imediato.

H1: A terapia com estimulação elétrica nervosa transcutânea combinada com o laser de baixa intensidade não promove melhora no desempenho funcional através do alívio da dor na região abdominal em mulheres submetidas à cesárea no puerpério imediato.

5. MATERIAIS E MÉTODOS

5.1 Caracterização da pesquisa

A pesquisa será caracterizada como ensaio clínico randomizado e cego, que irá comparar quatro grupos com distribuição aleatória. O cegamento ocorrerá nas avaliações do desfecho.

5.2 Procedimentos Éticos

O estudo será realizado respeitando as normas éticas estabelecidas na resolução n° 466/12 do Conselho Nacional de Saúde para pesquisa envolvendo seres humanos, e a casuística fará parte de estudo analisado, o qual será submetido à Plataforma Brasil. Posterior à aprovação do Comitê de Ética em Pesquisa, será iniciada o estudo com a seleção da amostra conforme os critérios de inclusão e mediante a assinatura do Termo de Consentimento Livre e Esclarecido (Apêndice A). O responsável pelo estudo irá esclarecer todo o procedimento da pesquisa às voluntárias.

5.3 Populas*o e amostra

A população do estudo será composta por 88 mulheres no puerpério imediato submetidas à cesárea, maiores de 18 anos de idade, recrutadas por conveniência e admitidas na Maternidade Divino Amor, durante o período de janeiro a dezembro de 2022.

As participantes serão divididas igualmente de forma aleatória em GT (grupo submetido ao TENS), GTL (grupo submetido ao TENS combinado com o LBI), GCP ou grupo controle por placebo (grupo com aplicação TENS e Laser ambos desligados) e GC ou grupo controle (sem intervenção).

5.3.1 *Cálculo* **amostra*l***

No cálculo amostral, a amostra do estudo foi resultante de um processo de amostragem do tipo probabilística.

Foi proposto o n com significância de acordo com a fórmula de Miot (2011)[14], baseado no grau de dor na região da incisão cirúrgica (como referência a Escala Numérica da Dor graduada de 0 a 10 e apresentando redução de 2 pontos no score de graduação da dor) e desvio padrão da variável para cada grupo de 1,5 com diferença mínima de 2. O erro alfa foi de 0,05, potência de teste de 80% e para as perdas foi considerado 20%[7] (Figura 01).

$$n = (Sa^2 + Sb^2 + Sc^2 + Sd^2) \cdot \left(\frac{Z\alpha/2 + Z\beta}{d} \right)^2$$

Nota: n = tamanho da amostra do estudo para cada grupo. Sa, Sb, Sc e Sd = desvio padrão da variável em cada grupo. Za/2 = valor do erro alfa, equivalente a 1,96 (5%). Z§ = valor do erro, equivalente a 0,84 (20%). d = diferença mínima entre as médias.

Figura 01: Fórmula para cálculo amostral do estudo.

Para cada grupo, o n foi de 17 voluntárias. Acrescentando 20% de possíveis perdas amostrais, o n final equivalente foi de 22 mulheres para cada grupo. Dessa forma, o tamanho total da amostra dessa pesquisa será composta de 88 participantes.

5.3.2 Critérios elegibilidade

5.3.2.1 Critérios de Inclusâo

Como critério de inclusão, as voluntárias deverão apresentar entre 8 a 12 horas de pós parto cesárea; apresentar dor na escala numérica da dor a 3 na região da incisão cesariana[7]; sem intercorrências clínicas ou obstétricas; sem processo infeccioso; deverão receber mesmo tratamento medicamentoso (anti-inflamatório, analgésico e antigases) com igual intervalo de ingestão; puérpera com dificuldade de comunicação e expressão para responder os questionamentos da avaliação; não apresentarem distúrbios e/ou alterações neurológicas; não apresentarem distúrbios

e/ou alterações osteomioarticulares; ser sedentária na avaliação com o Questionário IPAQ.

5.3.1.2 Critérios de Exclusão

Serão excluídas da pesquisa as mulheres que não desistirem de realizar a intervenção com a TENS; mulheres que desistirem de realizar a intervenção com o LBI; voluntárias que desistirem de realizar o teste de caminha de 2 (dois) minutos durante o período de coleta; voluntárias que apresentarem durante o período da coleta instabilidade clínica e obstétrica.

5.4 Instrumentas^o

Escala Numérica da

Dor

A avaliação da dor de forma unidimensional será verificada através da Escala Numérica da Dor[7 12] (Anexo A). Esta escala permite o paciente classificar a dor em grau 0 (zero) a grau 10 (dez). Grau 0 ou ausência de dor está localizado na extremidade da esquerda e aumenta gradativamente até extremidade da direita, representada pelo limite extremo de dor ou grau 10 de dor. O comando verbal utilizado para avaliação com esse instrumento será por meio de descritores verbais (0 = sem dor; 1-3 = dor branda; 4-6 = dor moderada; 7-10 = dor severa).

A dor na região abdmoninal da paciente será avaliada com a Escala visual numérica da dor quando a paciente estiver em repouso e em movimento (completado 1 minuto da realização do TC2M).

Algômetro Digital

Para a avaliação do limiar de dor será utilizado um algômetro digital[7] Force Gage modelo WAGNER FDM (Figura 02), que se trata de um dispositivo constituído por um disco de borracha 1cm^2 ligado a um medidor de pressão, que apresenta valores em kgf/cm^2. A participante será encorajada a dizer “começou” quando a pressão com a ponta do aparelho começar a evocar dor (limiar), a pressão continuará até a voluntária dizer “pare”. A pressão será aplicada perpendicularmente no ponto médio da distância entre a cicatriz umbilical e ferida operatória tendo como referência a linha alba (Figura 02). Para realizar a avaliação com o Algômetro Digital,

o disco de borracha será revestido com papel filme e higienizado com álcool 70%, sendo repetido o procedimento para cada voluntária da pesquisa.

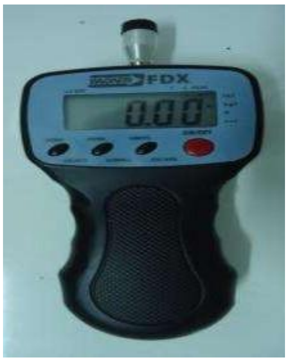

Figura 02: Algômetro Digital.

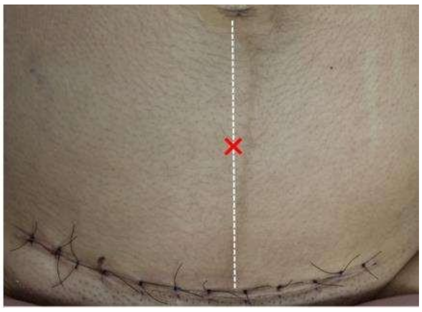

Figura 03: Local de avaliação do limiar da dor em mulheres submetidas a cesárea.

<u>Escala de Percepção Global de Mudança (PGIS)</u>

No estudo será feita avaliação da percepção do paciente em relação à intervenção da terapia combinada de TENS com LBI. Para essa avaliação será utilizado a PGIS (Anexo B), este é um instrumento validado na versão portuguesa fácil e rápido de ser aplicado com capacidade de medir a percepção de mudança do estado de saúde dos indivíduos quando submetidos a intervenções e determinar diferenças mínimas clinicamente importantes de instrumentos de avaliação da dor, função física e qualidade de vida[14].

A PGIS é uma medida unidimensional na qual permite os indivíduos classificarem a melhoria da sintomatologia dolorosa associando à intervenção através de uma escala de sete itens, distribuídos da seguinte forma: 1 = sem alterações; 2 = quase na mesma; 3 = ligeiramente melhor; 4 = com algumas melhorias; 5 = moderadamente melhor; 6 = melhor; 7 = muito melhor.

Medida de Independência Funcional (MIF)

No estudo será feita avaliação de independência funcional do paciente em relação à intervenção combinada da TENS com LBI. Para essa avaliação será utilizado a Medida de Independência Funcional (MIF), este é um instrumento validado para o português. A escala MIF adaptada classifica o paciente em sua habilidade para executar uma atividade independente e sua necessidade de assistência, quantificando a necessidade de ajuda do paciente[15].

A escala MIF adaptada[15] é organizada em três categorias: cuidados pessoais, mobilidade/transferências e locomoção. As atividades funcionais de cada item são pontuadas em graus de dependência, com escore máximo de 7 (independência completa) e mínimo de 1 (total necessidade de assistência), estabelecendo uma variação possível no resultado total de 11 a 77. Essa pontuação é estipulada pela escala, por meio de seu equivalente em funcionalidade de acordo com o Quadro 01. Para avaliar a perda funcional utiliza-se a seguinte equação: Perda Funcional% = (escore funcional momento inicial - escore funcional momento final) / (escore funcional momento inicial) x 100%.

Quadro 01: Nível de Funcionalidade para cada item da Escala

Nível	Equivalente em funcionalidade
7	Independência comp1eta: toda tarefa que envolve uma atividade, é realizada de rorma segura, sem modificações ou recursos auxiliares, dentro de um tempo razoável.
6	Independência modificada: capaz de realizar tarefas com recursos auxiliares, necessitando de mais tempo, porém realiza de forma segura e totalmente independente.
5	Supervisão: sujeito necessita somente supervisão ou comandos verbais ou modelos para realizar a tarefa sem a necessidade de contato ou a ajuda é somente para preparo da tarefa quando necessário.
4	Mínima assistência: necessita uma mínima quantidade de assistência, um simples tocar, possibilitando a execução da atividade (realiza 75% do esforço necessário na tarefa).
3	Moderada assistência: necessita uma moderada quantidade de assistência, mais do que simplesmente tocar, (realiza 50% do esforço necessário na tarefa).
2	Máxima assistência: utiliza menos que 50% do esforço necessário para completar a tarefa, mas não necessita auxílio total.
1	Total assistência: assistência total é necessária ou a tarefa não é realizada. Utiliza menos que 25% do esforço necessário para realizar a tarefa.

Fonte: 15”

Teste de caminhada de 2 minutos (TC2M)

No estudo será feita avaliação realizando o teste de caminhada de dois minutos que é utilizado para avaliar desempenho de marcha[12].

Para essa avaliação a paciente será convidada a realizar uma caminhada no corredor de 30 metros durante 2 minutos. A marcação do percurso será feita com uma fita adesiva colocada no chão e o tempo será marcado com o cronômetro digital. As voluntárias do estudo serão encorajadas a caminhar no ritmo mais rápido que conseguir sem correr, respeitando os seus limites. O examinador ficará atrás da paciente em torno de ^{1}7 (meio) metro para garantir segurança. Serão realizadas 2 tentativas práticas e um teste de caminhada de dois minutos final para registro na avaliação. Entre os testes são dados 10 minutos de intervalo para descanso da paciente[16,17]

Ao final do teste será feito a medição da distância percorrida em metros pelo paciente.

Questionário IPAQ (Questionário Internacional de Atividade Física)

Para avaliação do nível de atividade física será utilizado o IPAQ (Anexo C). Esse questionário é validado no Brasil e constitui de índice que classifica o indivíduo como muito ativo, ativo, irregularmente ativo e sedentário.

Para ser considerado sedentário, o indivíduo não realizou nenhuma atividade física por pelo menos 10 minutos durante a semana[18].

Laser de Baixa Intensidade (LBI)

Para a intervenção combinada, com objetivo de promover alívio da dor na região da incisão cesariana, será utilizado o LBI da marca DMC Therapy XT que possui comprimento de onda de 660nm (laser vermelho)[7], potência de 100 mW e óculos de proteção para o terapeuta e para a paciente.

Estimulação Elétrica Nervosa Transcutânea

Para a intervenção combinada, com objetivo de promover alívio da dor na região da incisão cesariana, será utilizado o equipamento Neurodyn II da IBRAMED que é utilizado para aplicação da corrente TENS[1] via eletrodos em contado direto com a paciente.

5.5 Procedimentos para a coleta

As voluntárias serão distribuídas aleatoriamente em quatro grupos: GT, GTL, GCP e GC. A randomização ocorrerá pelo site *randomization.com* estabelecendo um número para cada voluntária e será colocado em um envelope branco numerado e fechado. O envelope será aberto pelo Pesquisador 1 (responsãvel pela

randomização e que irá identificar o grupo da mulher para controle e intervenção), Pesquisador 2 (responsável pela avaliação das voluntárias do estudo), e Pesquisador 3 (responsável pela aplicação das duas sessões com o TENS e LBI)

Se durante o estudo houver exclusão de pacientes, as mesmas serão substituídas por outras automaticamente utilizando a mesma tabela de randomização.

Seguindo os critérios de elegibilidade da pesquisa, todas as voluntárias assinarão o TCLE e será preenchido o formulário de identificação da puérpera (Apêndice B) durante a Avaliação 1 (AV1) que ocorrerá entre 8 a 12 horas após o parto e que será composta por avaliação da dor com a Escala Numérica da Dor e Algometria, ambos com a paciente em repouso; Avaliação com o IPAQ; e, avaliação da independência funcional com a Escala de MIF. Após dez minutos do término da AV1, será realizada a intervenção com o GCP, GT e GTL.

Entre 20 a 24 horas após AV1, todas as puérperas do estudo se submeterão à AV2 que será composta por avaliação da dor com a Escala Numérica da Dor e Algometria, ambos em repouso; avaliação da dor com a Escala Numérica da Dor durante a realização do Teste de Caminhada de 2 minutos quando a paciente estiver completado 1 minuto do teste; avaliação da melhora pós intervenção através da Escala de Percepção Global de Mudança; e, avaliação da independência funcional com a Escala de MIF e Teste de Caminhada de 2 minutos. Após dez minutos do término da AV2, será realizado a segunda sessão da terapia TENS no GT e a segunda sessão da terapia combinada TENS com LBI no GCP e GTL.

Entre 44 a 48 após AV1, todas as voluntárias realizarão a Avaliação 3 (AV3) que utilizará os mesmos instrumentos de avaliação da AV2.O Pesquisador 2 será cego e ficará responsável em realizar a AV1, AV2 e AV3.

Nas avaliações serão considerados: dados sociodemográficos da paciente (nome, endereço, idade, nível educacional, estado civil, doenças associadas, infecção por coronavírus); dados obstétricos (idade gestacional, paridade, data do parto, tempo de pós parto); dados clínicos (avaliação da dor pela Escala Numérica da Dor, avaliação pelo Algômetro Digital, avaliação da percepção de melhora da dor através da Escala de Percepção Global de Melhora, Avaliação com IPAQ, Escala de Medida de Independência Funcional e Teste de Caminhada de 2 minutos).

Protocolo de Intervenção da TENS:

O protocolo de intervenção utilizará como referência o estudo de Kasapoglu et al (2020)[10]. A TENS será com frequência de 100Hz, largura de pulso de 75ps aplicado em duas sessões (30 minutos cada sessão) com intervalo de 20 a 24 horas entre as sessões no pós operatório imediato.

A intervenção TENS fará uso de dois eletrodos borracha para passagem da corrente que estarão em contato com a pele da paciente higienizados com álcool 70%, com gel condutor e serão posicionados acima e abaixo da feriada operatória. Os parâmetros da terapia TENS serão utlizados para o grupo GT e na terapia combinada no GTL. O posicionamento da paciente será em decúbito dorsal em posição neutra.

Protocolo de Intervenção do LBI:

O protocolo de intervenção utilizará como referência o estudo de Araújo et al (2019)[7]. O LBI será com comprimento de onda de 660nm (vermelho) em modo contínuo e realizado em duas sessões com intervalo de 20 a 24 horas. Os parâmetros da terapia Laser de Baixa Intensidade estão descritos na Tabela 01.

A técnica será de forma pontual sem contato e aplicado perpendicular à pele na linha da incisão cesariana[7]. O número de pontos a serem aplicados irá depender da extensão da ferida operatória, respeitando a distância de 1cm entre os pontos de aplicação (Figura 06). Para realizar a aplicação com o LBI, a caneta do aparelho deverá ser higienizada com álcool a 70%, no qual esse procedimento se repetirá para a intervenção em cada voluntária. Será feita assepsia com álcool a 70% antes e depois da intervenção na região da ferida operatória. O leito da paciente deverá estar exclusivamente presente o terapeuta e a paciente que farão uso de óculos de proteção específico do aparelho. O posicionamento da paciente será em decúbito dorsal em posição neutra.

Para a aplicação do LBI o mesmo será realizado de forma combinada com o TENS, sendo aplicados no grupo GTL

Tabela 01: Parâmetros do protocolo do LBI.

Parâmetros	Valores
Comprimento de onda	660nm (vermelho)

Modo de emissão	Modo contínuo
Potência	100Mw
Dose	2J/cm²
Energia	0,12J
Tempo de aplicação por ponto	4 segundos

Fonte: 7

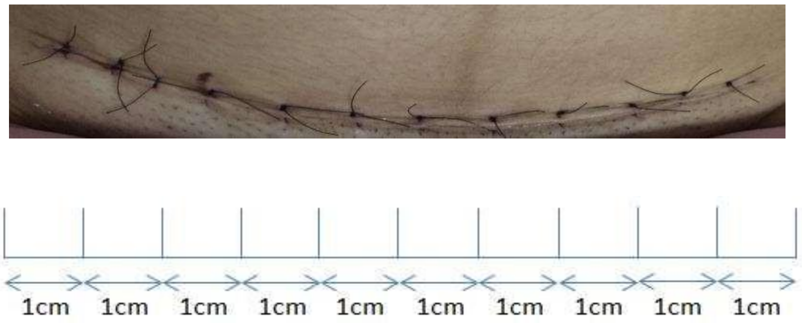

Figura 06: Irradiação do LBI com distância de 1cm entre os pontos de aplicação.

5.5.1 Dias e horários das coletas

As coletas serão realizadas no horário entre 07h às 10h, em três dias consecutivos (Quartas, Quintas e Sextas). Todas as coletas serão realizadas nos alojamentos conjunto de pós parto da Maternidade Divino Amor (Parnamirim/RN).

5.6 Análise estatística

Para análise estatística, os dados serão analisados utilizando os software SPSS 20.0 *(Statistical Package for the Social Sciences)* para Windows 23.0, atribuindo um nível de significância < 5%.

Inicialmente, será feita uma análise descritiva das variáveis do estudo por meio de medidas de tendência central e dispersão com a finalidade de se caracterizar a amostra que será exibida em forma de tabela com suas respectivas médias e desvio padrão de acordo com o teste de normalidade Kolmogorov-Sminorv. Para comparações de médias entre grupos (GC, GCP, GT e GTL) será utilizado o teste ANOVA, verificadas prováveis diferenças aplicar-se-á o teste de post hoc de Tukey.

5.7 Fluxograma do estudo

As etapas da pesquisa estão demonstradas no fluxograma abaixo, de acordo com o procedimento metodológico proposto no estudo.

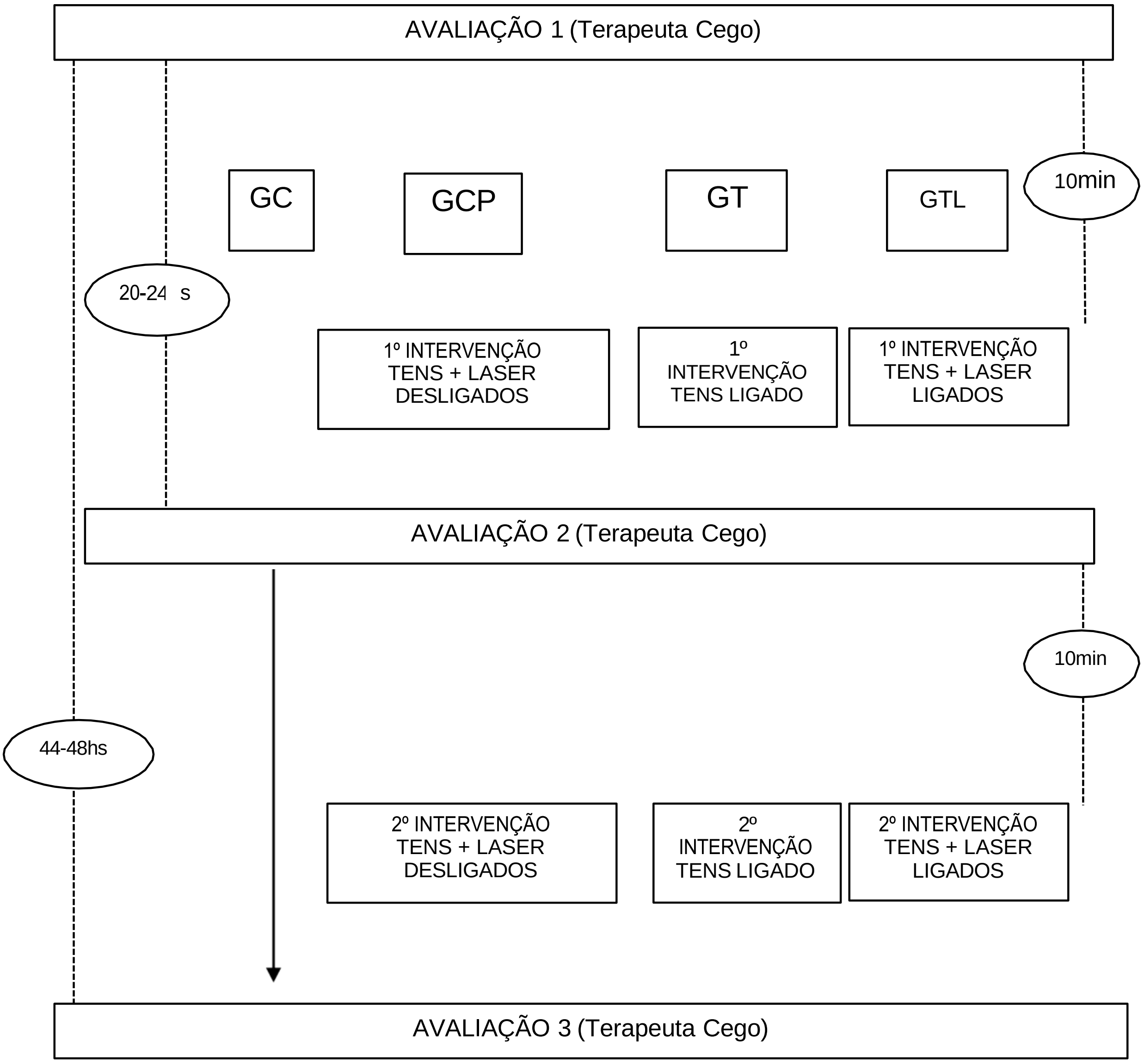

6. CRONOGRAMA

	2021<br>ii<br>semestre	2022<br>i<br>semestre	2022<br>ii<br>semestre	2023<br>i<br>semestre	2023<br>ii<br>semestre
Atualização Bibliográfica	X	X	X	X	
Submissão do Projeto ao comitê de ética e pesquisa	X				
Coleta dos Dados		X	X		
Análise dos Dados				X	X
Qualificação					X
Defesa					X
Submissão de Artigo					X

7. RESULTADOS ESPERADOS E PERSPECTIVAS PARA O AVANÇO DO CONHECIMENTO

Com o estudo é esperado verificar melhor desempenho funcional posterior aplicação de duas sessões da terapia combinada de TENS com LBI para quadro álgico na região da incisão cesariana. A pesquisa permitirá aos fisioterapeutas e comunidade acadêmica de fisioterapia, que a terapia combinada da TENS com LBI seja utilizada como recursos fisioterapêuticos em pacientes submetidas à cesárea que se encontrem no puerpério imediato, assim como favorecerá evidência cientifica para aplicabilidade da terapia combinada no alívio da sintomatologia dolorosa com melhora no desempenho funcional em novos estudos.

8. RISCOS E BENEFÍCIOS

Esta pesquisa tem riscos mínimos por se tratar de avaliação e tratamentos não invasivos. Esta pesquisa tem riscos mínimos por se tratar de avaliação e tratamentos não invasivos. A voluntária poderá apresentar dor z3 na Escala Numérica da Dor e assim ter receio durante a avaliação com algômetro, sendo reforçado pelo pesquisador que o exame de algometria objetiva avaliar a percepção ao incomodo da pressão e não para aumentar a dor. Outro possível risco será de infecção no local do corte da cesárea, mas essa possibilidade é mínima já que será realizada a assepsia antes e depois da aplicação com laser de baixa intensidade e a estimulação elétrica nervosa transcutânea no local dos eletrodos. Pode haver possibilidades de queda ao realizar o Teste de Caminha de 2 minutos, porém durante esse procedimento o terapeuta ficará próximo para maior segurança durante essa avaliação. Poderá também ter algum questionamento sobre a intervenção, a utilização do recurso de estimulação elétrica nervosa transcutânea, que será utilizada caso a voluntária faça parte do grupo intervenção, consiste em técnica com transmissão de corrente elétrica e por essa razão poderá ocorrer riscos de choques mas o aparelho se encontrará calibrado para realização do procedimento, sendo o aparelho preparado e testado o funcionamento antes da intervenção e durante o uso será monitorado pelo terapeuta com atenção para não ter contato com líquidos próximo ao aparelho ligado. O recurso da TENS também poderá permitir algum incômodo na pele por gerar sensações de formigamento causada pelo estímulo da corrente elétrica, porém a intensidade será de acordo com o limite permitido pelas voluntárias, sendo iniciado com intensidade zero e aumentando gradativamente. Outro risco que poderá surgir decorrente da aplicabilidade do TENS é que você poderá apresentar irritação da pele no local de colocação dos eletrodos, para essa situação será feito monitoramento durante a sessão, em caso de irritação a terapia será suspendida e será feito limpeza da área no local dos eletrodos. Poderá também ter algum questionamento sobre a intervenção. Pode haver possibilidades de queda ao realizar o Teste de Caminha de 2 minutos, porém durante esse procedimento, o terapeuta ficará próximo da paciente para maior seguran0a durante essa avaliação. A contaminação pelo coronavírus pode existir mas todos os protocolos de segurança para evitar a contaminação serão utilizados como higienização das mãos, uso de

luvas, uso de máscaras e os terapeutas responsáveis pela avaliação e intervenção estarão vacinados.

Poderá também ter algum questionamento sobre a intervenção. O pesquisador reforçará os benefícios com a pesquisa realizando explicação de todas as etapas da pesquisa enfatizando que é esperado que a aplicação do laser de baixa intensidade com a TENS diminua a dor do pós-parto cesáreo, melhorando o desempenho na realização de atividades que exijam movimentos nos primeiros dias pós o parto e a importância da pesquisa para qualidade de vida e atenção à saúde.

9. ORÇAMENTO

ITEM	**QUANTIDADE**	**VALOR UNITÁRIO (R$)**	**TOTAL (R$)**
Álcool a 70%	3 litros	5,00	15,00
Luvas	3 caixas	15,00	45,00
Lençol de Papel	3 Rolos	15,00	45,00
Impressões	250	0,10 (folha)	45,00
Envelopes	300	0,40	120,00
Algômetro Digital*	1	Disponível	Disponível
Laser de Baixa Intensidade*	1	Disponível	Disponível
TENS”	1	Disponível	Disponível
Cronometro digital*	1	Disponível	Disponível
Fita adesiva	3 unidades	7,00	21,00
		**Total**	**291,00**

Os itens marcados (*) já se encontram disponíveis com os pesquisadores, não sendo necessário custo para os mesmos.

10. REFERÊNCIAS

1 KASAPOĞLU I, KASAPOĞLU AKSOY M, ÇETINKAYA DEMIR B, ALTAN L. The efficacy of transcutaneous electrical nerve stimulation therapy in pain control after cesarean section delivery associated with uterine contractions and abdominal incision. Turk J Phys Med Rehab 2020;66(2):169-175.

2 LAVAND'HOMME P. Postoperative cesarean pain: real but is it preventable? Curr Opin Anaesthesiol. 2018;31:262-267.

3 BORGES N C, PEREIRA L V, DE MOURA L A, SILVA T C, PEDROSO C F. Predictors for Moderate to Severe Acute Postoperative Pain after Cesarean Section. Pain Research and Management. Pain Res Manag. 2016; 2016:5783817.

4 SOUSA L, PITANGUI A C R, GOMES F A, NAKANO A M S, FERREIRA C H J. Measurement and characteristics of post-cesarean section pain and the relationship to limitation of physical activities. Acta Paul Enferm 2009;22:741-747.

5 PEREIRA T R C, SOUZA F G, BELEZA A C S. Implications of pain in functional activities in immediate postpartum period according to the mode of delivery and parity: an observational. Brazilian Journal of Physical Therapy 2017;21(1):37-43.

6 CRESSWELL B M C et al. Pregnancy and Childbirth. Measurement of maternal functioning during pregnancy and postpartum: findings from the cross-sectional WHO pilot study in Jamaica, Kenya, and Malawi. (2020) 20:518.

7 ARAÚJO A M P H, SENA K R R, FILHO E M S, PEGADO R, MICUSSI M T A B C. Low-level laser therapy improves pain in postcesarean section: a randomized clinical trial. Lasers in Medical Science, 2019.

8 POURSALEHAN S, NESIOONPOUR S, AKHONDZADEH R, MOKMELI S. The Effect of Low-Level Laser on Postoperative Pain After Elective Cesarean Section. Anesth Pain Med. 2018;8(6):e84195

9 KAYMAN-KOSE S, ARIOZ D T, TOKTAS H, KOKEN G, KANAT-PEKTAS M, KOSE M, YILMAZER M. Transcutaneous electrical nerve stimulation (TENS) for pain control after vaginal delivery and cesarean section. J Matern Fetal Neonatal Medicine. 2014:1—4.

10 FABRE HSC et al. Anti-inflammatory and analgesic effects of low-level laser therapy on the postoperative healing process J Phys. Ther. Sci. 2015; 27(6)

11 PENG W W, TANG Z Y, ZHANG F R, LI H, KONG Y Z, IANNETTI GD, HU L.Neurobiological mechanisms of TENS-induced analgesia. Neurolmage 195 (2019) 396—408.

12 ELBOIM-GABYZON M, NAJJAR S A, SHTARKER H. Effects of transcutaneous electrical nerve stimulation (TENS) on acute postoperative pain intensity and mobility after hip fracture: A doubleblinded, randomized trial. Clinical Interventions in Aging 2019:14 1841—1850.

13 MIOT H A. Tamanho da amostra em estudos clínicos e experimentais. J Vasc Bras. 2011;10:275-278.

14 DOMINGUES L, CRUZ E. Adaptação Cultural e Contributo para a Validação da Escala Patient Global Impression of Change. Ifisionline 2011; 2(1).

15 BORGES J B C, FERRERA D L M P, CARVALHO S M R, MARTINS A S, ANDRADE R R, SILVA M A M. Avaliação da intensidade de dor e da funcionalidade no pós-operatório recente de cirurgia cardíaca. Braz J Cardiovasc Surg 2006; 21(4): 393-402.

16 SELMAN J P R, CAMARGO A A, SANTOS J, LANZA F C, CORSO S D. Reference Equation for the 2-Minute Walk Test in Adults and the Elderly. Respiratory care. 2014: 4: 57.

17 BOHANNON R W, WANG Y, GERSHON R C, Reliability, validity and minimal detectable change of 2-min walk test and 10-m walk test in frail older adults receiving day care and residential care. Archives of Physical Medicine and Rehabilitation 2015;96:472-7

18 MATSUDO S, ARAÚJO T, MARSUDO V, ANDRADE D, ANDRADE E, OLIVEIRA L C, BRAGGION G. Questionário internacional de atividade física (IPAQ): estudo de validade e reprodutibilidade no Brasil. Rev. bras. ativ. fís. saúde; 6(2): 05-18, 2001.

APÊNDICE

APÊNDICE A - TERMO DE CONSENTIMENTO LIVRE E ESCLARECIDO (TCLE)

UNIVERSIDADE FEDERAL DO RIO GRANDE DO NORTE
CENTRO DE CIÊNCIAS DA SAÚDE
DEPARTAMENTO DE FISIOTERAPIA

TERMO DE CONSENTIMENTO LIVRE E ESCLARECIDO (TCLE)

Esclarecimentos

Este é um convite para você participar da pesquisa: **“INFLUÊNCIA NO DESEMPENHO FUNCIONAL DA TERAPIA COMBINADA DE LASER E TENS NA INCISÃO CESARIANA COM QUADRO ÁLGICO: ensaio clínico randomizado"** que tem como pesquisadora responsável prof° Dra. Patrícia Angélica de Miranda Silva Nogueira.

Essa pesquisa pretende de avaliar o efeito do tratamento da estimulação elétrica nervosa transcutânea combinada com o laser de baixa intensidade para alívio da dor no local do corte cirúrgico da cesárea e consequentemente melhorar o desempenho na realização de atividades que exijam mobilidade durante a internação hospitalar.

O estudo será composto por quatro grupos de voluntárias. Em dois grupos, serão feitas duas sessões utilizando TENS e laser, sendo um grupo com os aparelhos desligados e no outro com os aparelhos ligados. Um terceiro grupo será submetido apenas ao TENS. Existirá um grupo da pesquisa que não será feita a aplicação de nenhum recurso de tratamento.

Caso decida participar você poderá fazer parte de um dos grupos do estudo e sua participação no grupo será feita através de um sorteio. Durante sua permanência no hospital, você será avaliada três vezes: primeira avaliação entre 8-12 horas após o parto, segunda avaliação entre 20-24 horas após o parto e terceira avaliação entre 44-48 horas após o parto.

Rubrica do Participante/Responsável Legal:	Rubrica do Pesquisador:

Durante sua permanência no hospital, você será avaliada três vezes: primeira avaliação entre 8-12 horas após o parto, segunda avaliação entre 20-24 horas após o parto e terceira avaliação entre 44-48 horas após o parto.

Nas avaliações serão considerados: dados sociodemográficos (nome, endereço, idade, nível educacional, estado civil, doenças associadas, infecção por coronavírus); dados obstétricos (idade gestacional, paridade, data do parto, tempo de pós parto); dados clínicos (informações sobre a dor como também melhora ou piora após terapia combinada do TENS com o laser, desempenho funcional para realizar movimentos).

Para avaliar a dor, será feito um exame chamado de algometria que será realizado com um aparelho que tem uma ponta de borracha. Durante esse exame você irá dizer “começou” quando você sentir dor decorrente da pressão feita pela ponta do aparelho, porém a pressão continuará até você dizer “pare”. Além desse exame, a dor será avaliada usando uma Escala Visual Numérica composta de números (0 a 10), onde 0 (Zero) não há dor e 10 (dez) dor máxima.

Também será avaliado melhora ou piora após terapia combinada do TENS com o laser; e, avaliação da independência funcional com a utilização de uma Escala e Teste de Caminhada de 2 minutos que será de acordo com seu limite.

Caso faça parte dos grupos que irá usar laser, esse aparelho será aplicado na região acima do corte cirúrgico. Este aparelho possui uma caneta que estará higienizada com álcool 70% e não será realizado contato direto na pele. Será necessário que você e o pesquisador estejam de óculos de proteção próprios para uso do aparelho. O seu leito deverá estar com as cortinas fechadas, estando presente apenas você e o terapeuta no momento da aplicação do laser.

Para a avaliação o tempo será em média 30 minutos e para o procedimento terapêutico o tempo será em média de 40 minutos.

Como esta pesquisa existirá possibilidade de gravação de vídeo e registro fotográfico será solicitado consentimento através de um formulário de todas as voluntárias. Informo que o pesquisador garantirá a realização da pesquisa em ambiente adequado e reservado para garantir a privacidade do participante.

Durante a realização da pesquisa poderão ocorrer eventuais desconfortos e possíveis riscos por se tratar de avaliação e tratamentos não invasivos.

Rubrica do Participante/Responsável Legal:	Rubrica do Pesquisador:

Você pode apresentar dor a3 na Escala Numérica da Dor e assim ter receio durante a avaliação com algômetro, porém o exame de algometria objetiva avaliar a percepção ao incomodo da pressão e não para aumentar a dor. Outro possível risco será de infecção no local do corte da cesárea, mas essa possibilidade é mínima já que será realizada a assepsia antes e depois da aplicação com laser de baixa intensidade e a estimulação elétrica nervosa transcutânea no local dos eletrodos. Pode haver possibilidades de queda ao realizar o Teste de Caminha de 2 minutos, porém durante esse procedimento o terapeuta ficará próximo para maior segurança durante essa avaliação. Poderá também ter algum questionamento sobre a intervenção, a utilização do recurso de estimulação elétrica nervosa transcutânea, que será utilizada caso você faça parte do grupo intervenção, consiste em técnica com transmissão de corrente elétrica e por essa razão poderá ocorrer riscos de choques mas o aparelho se encontrará calibrado para realização do procedimento, sendo o aparelho preparado e testado o funcionamento antes da intervenção e durante o uso será monitorado pelo terapeuta com atenção para não ter contato com líquidos próximo ao aparelho ligado. O TENS também poderá permitir algum incômodo na pele por gerar sensações de formigamento causada pelo estímulo da corrente elétrica, porém a intensidade será de acordo com o limite permitido por você, sendo iniciado com intensidade zero e aumentando gradativamente, você também poderá apresentar irritação da pele no local de colocação dos eletrodos, para essa situação será feito monitoramento durante a sessão, em caso de irritação a terapia será suspendida e será feito limpeza da área no local dos eletrodos. A contaminação pelo coronavírus pode existir mas todos os protocolos de segurança

para evitar a contaminação serão utilizados como higienização das mãos, uso de luvas, uso de máscaras e os terapeutas responsáveis pela avaliação e intervenção estarão vacinados.

Como benefícios da pesquisa você poderá melhorar o desempenho para realizar atividades que exijam movimentos nos primeiros dias de pós parto. Caso você esteja no grupo que não realizou a intervenção, você estará contribuindo para a ciência, trazendo respostas para possíveis novas terapias.

Rubrica do Participante/Responsável Legal:	Rubrica do Pesquisador:

Em caso de complicações ou danos à saúde que você possa ter relacionado com a pesquisa, compete ao pesquisador responsável garantir o direito à assistência integral e gratuita, que será prestada com tratamento fisioterapêutico e encaminhamentos médicos.

Durante todo o período da pesquisa você poderá tirar suas dúvidas ligando para a pesquisadora responsável Dra. Patrícia Angélica de Miranda Silva Nogueira, pelo contato: (84) 99173-9579.

Você tem o direito de se recusar a participar ou retirar seu consentimento, em qualquer fase da pesquisa, sem nenhum prejuízo para você.

Os dados que você irá nos fornecer serão confidenciais e serão divulgados apenas em congressos ou publicações científicas, sempre de forma anônima, não havendo divulgação de nenhum dado que possa Ihe identificar. Esses dados serão guardados pelo pesquisador responsável por essa pesquisa em local seguro e por um período de 5 anos.

Alguns gastos pela sua participação nessa pesquisa, eles serão assumidos pelo pesquisador e reembolsado para vocês.

Se você sofrer qualquer dano decorrente desta pesquisa, sendo ele imediato ou tardio, previsto ou não, você será indenizado.

Qualquer dúvida sobre a ética dessa pesquisa você deverá ligar para o Comitê de Ética em Pesquisa UFRN - Lagoa Nova Campus Central (CEP Central/UFRN) — instituição que avalia a ética das pesquisas antes que elas comecem e fornece proteção aos participantes das mesmas — da Universidade Federal do Rio Grande do Norte, nos telefones (84) 3215-3135 ou (84) 9.9193-6266, e-mail cepufrn@reitoria.ufrn.br. Você ainda pode ir pessoalmente à sede do CEP, de segunda a sexta, das 08h00min às 12h00min e das 14h00min às 18h00min, na Rua das Artes, sin. Campus Central UFRN. Lagoa Nova. Natal/RN. CEP: 59075-000.

Este documento foi impresso em duas vias. Uma ficará com você e a outra com o pesquisador responsável Dra. Patrícia Angélica de Miranda Silva Nogueira.

Rubrica do Participante/Responsável Legal:	Rubrica do Pesquisador:

Consentimento Livre e Esclarecido

Após ter sido esclarecido sobre os objetivos, importância e o modo como os dados serão coletados nessa pesquisa, além de conhecer os riscos, desconfortos e benefícios que ela trará para mim e ter ficado ciente de todos os meus direitos, concordo em participar da pesquisa **"INFLUÊNCIA NO DESEMPENHO FUNCIONAL DA TERAPIA COMBINADA DE LASER E TENS NA INCISÃO CESARIANA COM QUADRO ÁLGICO:** ensaio **clínico randomizado" e** autorizo a divulgação das informações por mim fornecidas em congressos e/ou publicações científicas desde que nenhum dado possa me identificar.

____

Assinatura do participante da pesquisa

Impressão datiloscópica do participante

Declaração do pesquisador responsável

Como pesquisador responsável pelo estudo **"INFLUÊNCIA NO DESEMPENHO FUNCIONAL DA TERAPIA COMBINADA DE LASER E TENS NA INCISÃO CESARIANA COM QUADRO ÁLGICO: ensaio clínico randomizado",** declaro que assumo a inteira responsabilidade de cumprir fielmente os procedimentos metodologicamente e direitos que foram esclarecidos e assegurados ao participante desse estudo, assim como manter sigilo e confidencialidade sobre a identidade do mesmo.

Declaro ainda estar ciente que na inobservância do compromisso ora assumido infringirei as normas e diretrizes propostas pela Resolução 466/12 do Conselho Nacional de Saúde — CNS, que regulamenta as pesquisas envolvendo o ser humano.

Natal, ____/____/______

____

Dra. Patrícia Angélica de Miranda Silva Nogueira

APÊNDICE B - FICHA DE AVALIAÇÃO

UNIVERSIDADE FEDERAL DO RIO GRANDE DO NORTE

CENTRO DE CIÊNCIAS DA SAÚDE

DEPARTAMENTO DE FISIOTERAPIA

Código da voluntária:____________ Data da coleta: ____/____/_____

- Identificação

Nome:____________________________________Idade:__________________
Telefone:__________________________Hora do parto:__________________
Bairro:_____________________Cidade/Município:________________________

- Idade gestacional: _______Paridade: ______Trabalho de parto: ( )Sim( )Não
- Doenças Associadas:____
- COVID-19:

Teve COVID-19 ( ) Sim ( ) Não Quantas vezes? ____
Foi infectado antes ou durante a gravidez?_______
Em caso de infecção por COVID-19 durante a gestação, qual foi o trimestre de gestação? ( ) 1º Trimestre ( ) 2º Trimestre ( ) 3º Trimestre

- Empregada: ( ) Sim ( ) Não Tabagista: ( ) Sim ( )Não Parceiro: ( ) Sim ( )Não
- Nível Educacional: _______________________ Anos de estudo:_____________

Alfabetizada	SIM	NÃO
Primário	COMPLETO	INCOMPLETO
Fundamental	COMPLETO	INCOMPLETO
Médio	COMPLETO	INCOMPLETO
Superior	COMPLETO	INCOMPLETO

1. Algômetro digital: _______________
2. Teste de Caminhada de 2 minutos (TC2M):_______Dor (1 minuto TC2M):______
3. Medida de Independência Funcional:

Categorias	AV1	AV2	AV3
CUIDADOS PESSOAIS			
Alimentação			
Auto cuidado			
Banhar-se			
Vestir tronco superior			
Vestir tronco inferior			
Higiene íntima			
MOBILIDADE			
Cama/cadeira/cadeira de rodas			
Banheiro			
Banho chuveiro/banheira			
LOCOMOÇÃO			
Andar/cadeira de rodas			
Escadas			
TOTAL			

ANEXO

ANEXO A - ESCALA NUMÉRICA DA DOR

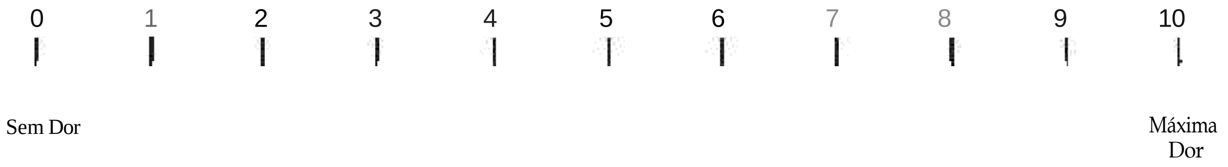

COMANDO VERBAL:

0 = sem dor

1-3 = dor branda

4-6 = dor moderada

7-10 = dor severa

ANEXO B - ESCALA DE PERCEPÇÃO GLOBAL DE MUDANÇA (PGIC versão Portuguesa)

Escala de Perceç*s^o Global de Mudança (PGIC versão Portuguesa)

Nome: ____ Data: ____________

Queixa principal: ____________________________________

Desde o início do tratamento nesta instituição, como é que descreve a mudança (se houve) nas LIMITAÇÕES DE ACTIVIDADES, SINTOMAS, EMOÇÕES E QUALIDADE DE VIDA no seu global, em relação à sua dor (selecione UMA opção):

Sem alterações (ou a condição piorou)	0	1
Quase na mesma, sem qualquer alteração visível		2
Ligeiramente melhor, mas, sem mudanças consideráveis		3
Com algumas melhorias, mas a mudança não representou qualquer diferença real	0	4
Moderadamente melhor, com mudança ligeira mas significativa	9	5
Melhor, e com melhorias que fizeram uma diferença real e útil	0	6
Muito melhor, e com uma melhoria considerável que fez toda a diferença		7

Adaptado e Validado por: Domingues, L. & Cruz, E. (2011)
Email: lucia.domingues@ess.ips.pt
Copyright 2004, Hurst, H. & Bolton, J.

ANEXO C — Questionário Internacional de Atividade Fisica (IPAQ versão curta)

Questionário Internacional de Atividade Fisica (IPAQ versão curta)

Nome. _____________
Data: _ Idade : Sexo: F) M (”›
Você traoaiha de 'o ma remunerada: ( ) Sim ( ) Não
Ouant3s 'horas você trabalha por dia: ________
Quantos anos completos vocé °stuóou: _
De form.a geral sun saúde astã:
(ExceletJte i) Muitc ho« ( ) Boa (, Regular ( ) Rulrn

Nós •stamos int4resSados em saber que .ipos de atividade física as pessoas taze.v co›TID ÇBrte Jo seu dia a dia. Este DFojeto faz parte de um orande estudo que está sendo teito em d.ferentes países ao redor do .unóo. 7r:as rospostas nos atudarao a entender que táo a’.ivos nôs somos em relação à pessoas õe outrss pai'ses. As perguntas asino relacionadas ao tempo que você gasta fazendo atividade tísica em uma semana NORMAL, USUAL uu HABITUAL. As perguntas \ac'uem as atividades que você faz no trabaiho, para ir ele um 'ugar a of !rc, nor lazer. por esporte. por exercício ou corno parte das suas at:vidaoes em casa ou no jardim. Suas resposlks S3o MUITO \mpsrtanles, Por fuvor resçonds oada questão mesmo que co.nsidere oue nâc seja ntivc. Obrigado peIn uma pariicigação !

PcYra rr,0ponder as questões \embre que:
° at vitJÂdes físicas VIGOROSAS súo aquelas qüe grectsam de um grande esforço físico e que fazem esperar MuITo m,vis fo u quo o normal
• atividades ^.isicas MODERADAS Süo aquo!ns qua prec‹s«m de algum esforço físico e qUe fazem rcspi• ra uM POUCO mais forte que o normal

Para rus¿onoer as pergunt‹vs pense somente nas atividades que você reaJ›z a oor oela menos 10 minutos çA? nuAp de corda vez:

ta. Em quarltofi Úias de umtt sorY\aiFIiT fiDFMüI, VOCÕ FOüliza a"i'7‹dades VIGOROSAS por q_eIo menos 1U minutos ron”i”ni n.e, como por ux0mp.u correr, *azer girústica aeróbica, jogar futebol. pedalar rápido na bicicleta. ¡•s•r basquete faze' serviços úomú stic0s pesados em casa, no quintal ou no jardim. car regar pesos e.eY'aãos ou qualo.uer at›viüa04 que 'aba você suar BASTANTE ou aumentem k\UJTO sua resp.'raçâo ou batimentcs do coreçãc.

dias _____Lor SEMANA ( ) N0nf\um

1b. Nm dias em quo voc6 faz essas atividades vigorosas por p.eq mene O minutos co tínuos, quanto tempo no iotai você gastu fczendu essa:e atividades por_cIig?
Carr. . _ _ Minutos:

2a. Em rjuztnto * dias de umr‹ s‹ mana norma.. VOCE LB3IÍZn atividades MODERADAS por ! menns 10 m.nu-los continuo . como por 4xrmplo ¿edalar Teve nn bicicleta nadar. dançar. fazer ginástica aeróbiCa leve. jogar vôlei reereaii\ o. ear regar USOS iGv”0.0, t42E"r serviços doméat!COS na cusa. no quintal ou no jardim como varrer. aspirar. cuidar do jardim. ou qualquer atividade que faça você suar .eve ou aumanteic moderadamente sua respiração ou batim0ntub do curaçao {POfl FAVOR NÃO tNCLMA CAMINHADA)

dias _____por SEMANA ( ) Nenhum

2b. Nos dias err› Que você faz assas atividades modsradas por po.Ic menos 1d m!nutos contar.uos quanto tempo ns total você game fazendo essas ajiviJades gozclJa?

horas' ______ Minutos: _

3s Em quantos.dias.de uma semana normal v 6 caminha par:pelo menos J0 minutos oont/nuos.em ca6a.ou no trabaiho, como lorma de transporte para ir:de.um lugaf para” outro, par:lazer, por.prazer ou como forma de 6xâK ÍGIO†

diaB _____por SEMAFiA ( ) Nenhum

3”b. Nos dita am que voó”6 caminha por D61O g'tBD0g 10 minutos oniiúuos quanto tempo no total gasa caminhando oor dla7

horas: ______Minutos: _____

4a. E8tas últimas pe@úntas aão em relação ao tempo que vocd gasta sentado ao iado no.trabalho,.em casa, na escola ou Faculdade a. durante: o tampo livra. Isto inclui o tempo que voc”i2 gas”ta sentado no escritô”rio au estudando; f82ando lx;4o de casa;vis'rlando aMi8••. I9ndo a &0ntado ou de'itadô assisôndo telavisâü.

ÕuantD”tampo por dia.você fica sentado em um dia da.semana*

horas: ______Minutos: _____

4”b. Oúdnto te'npo Dor dia voc”ê fica sentado no”fioal de semana†”

______ _____
