# Supplementary material for: TENS alone or combined with low-level laser therapy photobiomodulation for pain, functional capacity, and cardiorespiratory physiological variables after cesarean section: protocol for a randomized clinical trial
Source: PLoS One. 2025 Jun 20;20(6):e0325254. doi: 10.1371/journal.pone.0325254 (PMC12180720; doi:10.1371/journal.pone.0325254)
Supplement: S1 File — (ZIP) [file pone.0325254.s002.zip › Study complete_approved in english.pdf]

FEDERAL UNIVERSITY OF RIO GRANDE DO NORTE HEALTH
SCIENCES CENTER
GRADUATE PROGRAM IN PHYSICAL THERAPY

INFLUENCE ON THE FUNCTIONAL PERFORMANCE OF COMBINED LASER AND TENS THERAPY IN CESAREAN INCISION WITH PAIN: trial randomized clinical

Alane Macatrão Pires de Holanda Araújo Sena

NATAL - RN
2021

Alane Macatrão Pires de Holanda Araújo Sena

INFLUENCE ON THE FUNCTIONAL PERFORMANCE OF COMBINED THERAPY OF LASER AND TENS IN THE CESAREAN INCISION WITH PAIN: **test randomized clinical**

Project presented to the Graduate Program in Physical Therapy of the Federal University of Rio Grande do Norte for selection in the Doctorate in Physical Therapy.

Line of Research: Evaluation and intervention in Physiotherapy in the cardiovascular and respiratory systems.
Advisor: Profa. Dr. Patrícia Angélica de Miranda Silva Nogueira

NATAL - RN

2021

SUMMARY

SUMMARY 13

ABSTRACT 14

1. INTRODUCTION 9

2. JUSTIFICATION 11

3. OBJECTIVES 12

3.1 General objective 12

3.2 Specific objectives 12

4. HYPOTHESES 13

5. **MATERIALS** AND METHODS 14

5.1 Characterization of the research 14

5.2 Ethical Procedures 14

5.3 Population and sample 14

5.3.1 Sample calculation 15

5.3.2 Eligibility criteria 15

5.3.2.1 Inclusion criteria 15

5.3.1.2 Exclusion Criteria 16

5.4 Instrumentation 16

5.5 Procedures for collection 20

5.5.1 Days and times of collections 23

5.6 Statistical analysis 23

5.7 Study flowchart 24

6. SCHEDULE 25

7. EXPECTED RESULTS AND PERSPECTIVES FOR THE ADVANCEMENT OF THE KNOWLEDGE 26

8. RISKS AND BENEFITS 27

9. BUDGET 28

10. REFERENCES 29

APPENDIX 32

APPENDIX A - INFORMED CONSENT FORM (ICF) 32

APPENDIX B - EVALUATION SHEET 37

ATTACHMENT 38

APPENDIX A - NUMERICAL PAIN SCALE 38

ANNEX B - GLOBAL PERCEPTION OF CHANGE SCALE (PGIC PORTUGUESE VERSION) 39

ANNEX C - INTERNATIONAL PHYSICAL ACTIVITY QUESTIONNAIRE (IPAQ SHORT VERSION) 40

SUMMARY

Introduction: In the immediate puerperium of women undergoing cesarean section, the main complaint is pain in the abdominal region resulting from the postpartum surgical procedure, generating discomfort and impact on the necessary activities of the puerperal woman with the baby and movements in the bed itself. For a better quality of life of postpartum women who are in the first days postpartum, it is important that the physiotherapist professional performs a conduct acting on the main complaints considering the patient's clinical condition. LLLT and TENS are non-invasive resources that are being used in scientific research in studies aiming at analgesia, favoring applicability in clinical practices in isolation in puerperal women, but no studies were found in the literature that verified the effects of combined therapy of these resources in post-cesarean section women and their influence on functional performance. **Objective:** To evaluate the influence on functional performance of transcutaneous electrical nerve stimulation combined with low-level laser therapy in women undergoing cesarean section with pain. **Methods:** The research will be characterized as a randomized and blinded clinical trial. The sample will be composed of women in the immediate postpartum period after cesarean section, following the eligibility criteria proposed in the study and randomly distributed into four groups: control (CG), placebo control (GCP), group with TENS combined with low-level laser (GTL) and group with TENS application (GT). All groups will be submitted to three evaluations (data from the medical record, Global Perception of Improvement Scale, Numerical Pain Scale, the Digital Algometer, 2-minute Walk Test and Functional Independence Measure). The study will follow the protocol with non-contact 660nm Low Intensity Laser in the cesarean incision in two sessions combined with 100Hz TENS with electrodes above and below the surgical wound. In the study there will be a group that will be submitted only with TENS. In the statistical analysis, a descriptive analysis of the study variables will be performed according to the Kolmogorov-Sminorv normality test. For comparisons of means between groups, the ANOVA test will be used and if probable differences are verified, Tukey's post hoc test will be applied. **Expected results:** It is expected

to verify improvement in functional performance to perform activities that require movements after combined therapy of TENS with Low Intensity Laser in the cesarean incision, which will allow physiotherapists and the academic community to use this therapeutic resource in the target audience of this study, as well as favoring scientific evidence for applicability in pain symptoms in new research.

Keywords: Puerperium. Phototherapy. Electrotherapy. Functional physical performance.

ABSTRACT

Introduction: In the immediate puerperium of women undergoing cesarean section, pain in the abdominal region resulting from the postpartum surgical procedure is reported, causing discomfort and impact on the puerperal woman's necessary activities with the baby and movements in the bed itself. For a better quality of life for the mothers who are in the first days of postpartum, it is important that the physiotherapist perform a conduct acting on the main complaints considering the clinical condition of the patient. LBI and TENS are non-invasive resources that are being used in scientific research in studies aimed at analgesia, favoring applicability in clinical practices in isolation in puerperal women, but studies that verified the effects of combined therapy of these resources in postpartum women were not found in the literature. cesarean section and its influence on functional performance. Aim: Evaluate the influence on the functional performance of transcutaneous electrical nerve stimulation combined with Iow-intensity laser therapy in women undergoing cesarean with pain. **Methods:** The research will be characterized as a randomized and blind clinical trial. The sample will consist of women in the immediate post- cesarean postpartum period, following the eligibility criteria proposed in the study and randomly distributed into four groups: control (GC), placebo control (PCG), group with application of TENS combined with laser low intensity (GTL) and group with application of TENS (GT). AII groups will undergo three assessments (data from medical records, Global Perception of Improvement Scale, Numerical Pain Scale, Digital Algometer, 2-minute Walk Test and Functional Independence Measure). The study will follow the protocol with Low Intensity Laser of 660nm without contact in the cesarean incision in two sessions combined with TENS of 100Hz with electrodes

above and below the surgical wound. In the study there will be a group that will be submitted only with TENS. In the statistical analysis, a descriptive analysis of the study variables will be performed according to the Kolmogorov-Sminorv normality test. For comparisons of means between groups will be used the ANOVA test and verified probable differences will apply Tukey's post hoc test. **Expected Results:** It is expected to verify improvement in functional performance to perform activities that require subsequent movements combined with TENS therapy with Low Intensity Laser in the cesarean section, which will allow physiotherapists and the academic community to use this therapeutic resource in the target audience of this study, as well as favoring evidence for applicability in painful symptomatology in new research.

Keywords: Postpartum period. Phototherapy. Eletrotherapy. Physical Functional Performance.

1. INTRODUCTION

The postpartum cesarean section is characterized by the presence of complaints such as pain in the abdominal region and limitation of activities that require movement, especially in the first 24 hours after delivery1 [2 3]. Pain in the region of the cesarean section incision is considered a post-cesarean section morbidity for mother and baby, characterized as postoperative pain that is difficult to recover. Pain in the surgical wound is related to the inflammatory phase of the tissue repair process and thus results in difficulty in breastfeeding positions, newborn care and for activities such as sitting, standing, walking and hygiene

intimate4,5

A study carried out with the application of the WHODAS-12 questionnaire, used to assess the degree of difficulty in performing activities presented by the individual in the last 30 days, was applied to women in the postpartum period, and the presence of difficulty in mobility (such as standing and walking) with an impact on breastfeeding was verified in its results6.

In some studies, non-pharmacological resources are suggested in therapies for clinical conditions in the immediate puerperium (period from the first day to the tenth day after delivery) in women undergoing cesarean section to favor analgesia4 such as Low Intensity Laser (LLLT)' [8] and Transcutaneous Electrical Nerve Stimulation (TENS)[1] ^. LLLT consists of light emission and promotes analgesic effect in acute or chronic bone, muscle and tendon lesions, vasodilation and proliferation of microvessels, with a possible increase in the amount of oxygen in the tissue, epithelial, endothelial and fibroblastic proliferation, increased collagen synthesis and phagocytic activity, accelerating the tissue repair process, in addition to the release of cytokines that will reduce the inflammatory reaction10. In the case of TENS, it consists of an electric current that when used at high frequency (50 - 100Hz) promotes analgesia and its effects involve pain control systems through the activation of the endogenous opioid system and gate theory (the stimulation of large-caliber afferent nerve fibers and rapid conduction speed are transmitted to the spinal cord, where they temporarily block nociceptive pain stimuli to the

brain)[11.12]

In order to verify analgesic effects of LLLT therapy, it was verified in the study carried out with 88 women submitted to cesarean section randomly distributed into 4 groups (control, placebo, intervention with a dose of 2 J/cm2 and intervention with a dose of 4 J/cm2) by applying two sessions of LLLT with a wavelength of 660 nm (first session in 8-12 hours postpartum and the second session in 20-24 hours postpartum), reduction of pain in the surgical wound after intervention with perception of global improvement reported by the patient through the application of the Global Perception of Change Scale (PGIC Portuguese version). In this study, the LLLT with a dose of 2 J/cm2 presented, in its results, a greater effect size when compared to the other groups in the study7.

In the clinical trial carried out with 90 volunteers submitted to cesarean delivery and randomly distributed in two groups (control group and group submitted to TENS therapy) it was observed that the TENS therapy placed the electrodes below and above the cesarean incision using parameters with a frequency of 100Hz and pulse width of 75ps applied in two sessions (30 minutes each session) with an interval of 24 hours between sessions in the immediate postoperative period, It promotes improvement in the complaint of pain reported by puerperal women in the abdominal region associated with surgical incision and postpartum uterine contractions1.

It is reported in the literature that pain in the acute postoperative period influences mobility1, and it is observed that the use of TENS, with the objective of improving pain in patients undergoing surgical procedures, is suggestive as a therapy to favor better execution of activities that require movements in the acute phase by promoting pain relief12.

In view of the pain with limitation of movement in the first days after cesarean section combined with the effects of TENS found in the literature for analgesia with consequent improvement in the execution of movements and the scientific findings also about analgesia with the use of LLLT therapy, this study will have the purpose of verifying the possibility of applying TENS therapy combined with LLLT in the surgical wound of women undergoing cesarean section with complaints of pain in the improvement of functional performance after intervention during the immediate puerperium.

2. JUSTIFICATION

In the immediate puerperium of women undergoing cesarean section, the main complaint is pain in the abdominal region resulting from the postpartum surgical procedure, generating discomfort and impact on the necessary activities of the puerperal woman with the baby and movements in the Ieito4 [10].

For a better quality of life of postpartum women who are in the first days postpartum, it is important that the physiotherapist professional performs a conduct acting on the main complaints considering the patient's clinical condition. LLLT and TENS are non-invasive resources that are being used in scientific research in studies aimed at analgesia, favoring applicability in clinical practices in isolation in puerperal women, but no studies were found in the literature that verified the effects of combined therapy of these resources in post-cesarean section women.

In view of the above, it is necessary to verify the possibility of using the combined resources of LLLT and TENS therapy in the cesarean section incision for analgesia and its influence on the functional performance of patients, to strengthen evidence-based physical therapy and stimulate the realization of new studies related to the theme.

3. OBJECTIVES

3.1 General objective

To evaluate the influence on functional performance of transcutaneous electrical nerve stimulation combined with low-level laser therapy in women undergoing cesarean section with pain.

3.2 Specific objectives

- To assess pain in the abdominal region in women undergoing cesarean section through the numerical pain scale over time with the patient at rest and in motion;
- To verify the pain threshold in the abdominal region in women undergoing cesarean section over time through algometry;
- To evaluate the improvement of the clinical condition through the Global Perception of Change Scale in the first 24 hours and 48 hours postpartum;
- To assess functional independence through the Functional Independence Measurement Scale at 8-12h, 20-24h and 44-48h after delivery;
- Evaluate functional performance through the 2 (two) minute test in the first 24 hours and 48 hours after delivery;
- To compare intergroup functional performance over time.

4. CHANCE

HO: Therapy with transcutaneous electrical nerve stimulation combined with low-level laser promotes improvement in functional performance by relieving pain in the abdominal region in women undergoing cesarean section in the immediate postpartum period.

H1: Therapy with transcutaneous electrical nerve stimulation combined with low-level laser does not improve functional performance by relieving pain in the abdominal region in women undergoing cesarean section in the immediate puerperium.

5. MATERIALS AND METHODS

5.1 Characterization of the research

The research will be characterized as a randomized and blinded clinical trial, which will compare four groups with random distribution. Blinding will occur in outcome assessments.

5.2 Ethical Procedures

The study will be carried out respecting the ethical standards established in resolution No. 466/12 of the National Health Council for research involving human beings, and the casuistry will be part of the analyzed study, which will be submitted to Plataforma Brasil. After approval by the Research Ethics Committee, the study will begin with the selection of the sample according to the inclusion criteria and by signing the Informed Consent Form (Appendix A). The person responsible for the study will clarify the entire research procedure to the volunteers.

5.3 Populations and sample

The study population will consist of 88 women in the immediate puerperium undergoing cesarean section, over 18 years of age, recruited by convenience and admitted to the Divino Amor Maternity, during the period from January to December 2022.

Participants will be divided equally randomly into GT (group submitted to TENS), GTL (group submitted to TENS combined with LBI), GCP or placebo control group (group with TENS application and Laser both turned off) and CG or control group (no intervention).

5.3.1 Sample calculation l

In the sample calculation, the study sample was the result of a probabilistic sampling process.

The n was proposed with significance according to the formula of Miot (2011)[14], based on the degree of pain in the region of the surgical incision (as a reference to the Numerical Pain Scale graded from 0 to 10 and with a reduction of 2 points in the pain grading score) and standard deviation of the variable for each group of 1.5 with a minimum difference of 2. The alpha error was 0.05, test power was 80%, and losses were 20%[7] (Figure 01).

$$n = (Sa^2 + Sb^2 + Sc^2 + Sd^2) \cdot \left(\frac{Z\alpha/2 + Z\beta}{d} \right)^2$$

Note: n = study sample size for each groupo. Sa, Sb, Sc, and Sd = standard deviation of the variable in each group. Za/2 = alpha error value, equivalent to 1.96 (5%). Z§ = error value, equivalent to 0.84 (20%). d = minimum difference between the means.

Figure 01: Formula for sample calculation of the study.

For each group, the n was 17 volunteers. Adding 20% of possible sample losses, the final equivalent n was 22 women for each group. Thus, the total sample size of this research will be composed of 88 participants.

5.3.2 Eligibility criteria

5.3.2.1 Inclusion criteria

As an inclusion criterion, volunteers must present between 8 and 12 hours after cesarean delivery; presenting pain on the numerical pain scale A 3 in the region of the cesarean section7; no clinical or obstetric complications; without infectious process; they should receive the same drug treatment (anti-inflammatory, analgesic and antiques) with the same interval of intake; puerperal women with difficulty in communication and expression to answer the evaluation questions; have no neurological disorders and/or alterations; have no disorders

and/or musculoskeletal changes; being sedentary in the assessment with the IPAQ Questionnaire.

5.3.1.2 Exclusion Criteria

Women who do not give up on the intervention with TENS will be excluded from the research; women who give up on the intervention with the LBI; volunteers who give up performing the 2 (two) minute walk test during the collection period; volunteers who present clinical and obstetric instability during the collection period.

5.4 Instruments^o

Numerical Pain Scale

The assessment of pain in a unidimensional way will be verified through the Numerical Pain Scale7 [12] (Appendix A). This scale allows the patient to classify pain as grade 0 (zero) to grade 10 (ten). Grade 0 or no pain is located at the left end and gradually increases to the right extremity, represented by the extreme pain threshold or grade 10 pain. The verbal command used for evaluation with this instrument will be through verbal descriptors (0 = no pain; 1-3 = mild pain; 4-6 = moderate pain; 7-10 = severe pain).

Pain in the patient's abdominal region will be assessed with the Visual Numerical Pain Scale when the patient is at rest and moving (completed 1 minute of performing the 2MWT).

Digital Allometer

To assess the pain threshold, a digital algometer7 Force Gage model WAGNER FDM (Figure 02) will be used, which is a device consisting of a 1cm 2 rubber disc connected to a pressure gauge, which presents values in kgf/cm2. The participant will be encouraged to say "started" when the pressure with the tip of the device begins to evoke pain (threshold), the pressure will continue until the volunteer says "stop". Pressure will be applied perpendicularly at the midpoint of the distance between the umbilical scar and the surgical wound with reference to the linea alba (Figure 02). To perform the evaluation with the Digital Allometer,

The rubber disc will be coated with plastic wrap and sanitized with 70% alcohol, and the procedure will be repeated for each research volunteer.

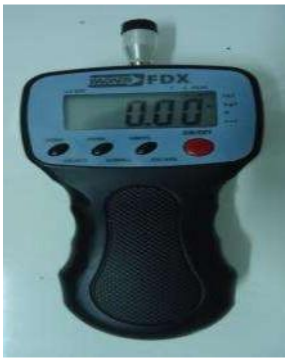

Figure 02: Digital Allometer.

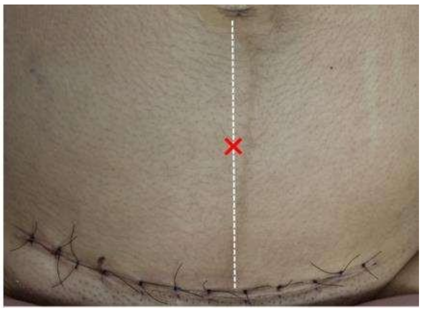

Figure 03: Site of pain threshold assessment in women undergoing cesarean section.

<u>Global Perception of Change Scale (PGIS)</u>

In the study, the patient's perception of the intervention of the combined therapy of TENS with LLLT will be evaluated. For this assessment, the PGIS (Appendix B) will be used, this is an instrument validated in the Portuguese version, easy and quick to be applied, with the ability to measure the perception of change in the health status of individuals when submitted to interventions and to determine minimum clinically important differences in pain assessment instruments, physical function and quality of life14.

The PGIS is a unidimensional measure in which it allows individuals to classify the improvement of pain symptoms by associating it with the intervention through a seven-item scale, distributed as follows: 1 = no changes; 2 = almost the same; 3 = slightly better; 4 = with some improvements; 5 = moderately better; 6 = better; 7 = much better.

Functional Independence Measure (MIF)

In the study, the patient's functional independence will be evaluated in relation to the combined intervention of TENS with LLLT. For this evaluation, the Functional Independence Measure (MIF) will be used, this is an instrument validated for Portuguese. The adapted FIM scale classifies the patient on their ability to perform an independent activity and their need for assistance, quantifying the patient's need for help15.

The adapted FIM scale15 is organized into three categories: personal care, mobility/transfers, and locomotion. The functional activities of each item are scored in degrees of dependence, with a maximum score of 7 (complete independence) and a minimum of 1 (total need for assistance), establishing a possible variation in the total result from 11 to 77. This score is stipulated by the scale, through its equivalent in functionality according to Chart 01. To assess functional loss, the following equation is used: Functional Loss% = (functional score at the beginning - functional score at the end of the moment) / (functional score at the beginning of the season) x 100%.

Table 01: Level of Functionality for each item of the Scale

Level	Equivalent in functionality
7	Complete independence: every task that involves an activity is carried out in a safe way, without modifications or ancillary features, within a reasonable time.
6	Modified independence: able to perform tasks with auxiliary resources, requiring more time, but performs safely and completely independently.
5	Supervision: subject only needs supervision or verbal commands or models to perform the task without the need for contact or help is only for task preparation when necessary.
4	Minimal assistance: requires a minimum amount of assistance, a simple touch, enabling the execution of the activity (performs 75% of the effort required in the task).
3	Moderate assistance: needs a moderate amount of assistance, more than simply touch, (performs 50% of the effort required in the task).
2	Maximum assistance: uses less than 50% of the effort required to complete the task, but does not needs full help.
1	Full assistance: Full assistance is required or the task is not performed. Uses less than 25% of the effort required to accomplish the task.

Source: 15"

2-minute walk test (2Mct)

In the study, an evaluation will be carried out using the two-minute walk test, which is used to assess gait performance12.

For this evaluation, the patient will be invited to walk in the 30-meter corridor for 2 minutes. The marking of the course will be done with an adhesive tape placed on the ground and the time will be marked with the digital stopwatch. The study volunteers will be encouraged to walk at the fastest pace they can without running, respecting their limits. The examiner will be around [17 (half)] meters behind the patient to ensure safety. 2 practice trials and a final two-minute walk test will be performed to register for the assessment. Between the tests, a 10-minute break is given to rest the

patient16,17

At the end of the test, the distance traveled in meters by the patient will be measured.

IPAQ Questionnaire (International Physical Activity Questionnaire)

To assess the level of physical activity, the IPAQ (Annex C) will be used. This questionnaire is validated in Brazil and is an index that classifies the individual as very active, active, irregularly active and sedentary.

To be considered sedentary, the individual did not perform any physical activity for at least 10 minutes during the week18.

Low Intensity Laser (LBI)

For the combined intervention, with the objective of promoting pain relief in the region of the cesarean section, the DMC Therapy XT brand LLLT will be used, which has a wavelength of 660nm (red laser)[7], power of 100 mW and protective glasses for the therapist and the patient.

Transcutaneous Electrical Nerve Stimulation

For the combined intervention, with the objective of promoting pain relief in the region of the cesarean section, IBRAMED's Neurodyn II equipment will be used, which is used to apply the TENS1 current via electrodes in direct contact with the patient.

5.5 Collection procedures

The volunteers will be randomly distributed into four groups: GT, GTL, GCP and CG. Randomization will take place through the website *randomization.com* establishing a number for each volunteer and will be placed in a numbered and closed white envelope. The envelope will be opened by Researcher 1 (responsible for the

randomization and that will identify the woman's group for control and intervention), Researcher 2 (responsible for the evaluation of the study volunteers), and Researcher 3 (responsible for the application of the two sessions with the TENS and LBI)

If patients are excluded during the study, they will be replaced by others automatically using the same randomization table.

Following the eligibility criteria of the research, all volunteers will sign the ICF and the puerperal woman's identification form (Appendix B) will be filled out during Assessment 1 (AV1) which will take place between 8 and 12 hours after delivery and which will consist of pain assessment with the Numerical Pain Scale and Algometry, both with the patient at rest; Evaluation with the IPAQ; and, evaluation of functional independence with the FIM Scale. Ten minutes after the end of AV1, the intervention will be performed with the GCP, GT and GTL.

Between 20 and 24 hours after AV1, all puerperal women in the study will undergo VA2, which will consist of pain assessment with the Numerical Pain Scale and Algometry, both at rest; pain assessment with the Numerical Pain Scale during the 2-minute Walk Test when the patient has completed 1 minute of the test; evaluation of post-intervention improvement through the Global Perception of Change Scale; and, evaluation of functional independence with the FIM Scale and 2-minute Walk Test. Ten minutes after the end of AV2, the second session of TENS therapy will be performed in the TG and the second session of combined TENS therapy with LLLT in the GCP and GTL.

Between 44 and 48 years after AV1, all volunteers will perform Assessment 3 (AV3) which will use the same assessment instruments as AV2. Researcher 2 will be blind and will be responsible for performing AV1, AV2 and AV3.

The evaluations will consider: the patient's sociodemographic data (name, address, age, educational level, marital status, associated diseases, coronavirus infection); obstetric data (gestational age, parity, date of delivery, postpartum time); clinical data (pain assessment by the Numerical Pain Scale, assessment by the Digital Algometer, assessment of the perception of pain improvement by the Global Perception of Improvement Scale, Assessment with IPAQ, Functional Independence Measure Scale and 2-minute Walk Test).

<u>TENS Intervention Protocol:</u>

The intervention protocol will use the study by Kasapoglu et al (2020)[10] as a reference. TENS will be with a frequency of 100Hz, pulse width of 75ps applied in two sessions (30 minutes each session) with an interval of 20 to 24 hours between sessions in the immediate postoperative period.

The TENS intervention will make use of two rubber electrodes for the passage of current that will be in contact with the patient's skin sanitized with 70% alcohol, with conductive gel and will be positioned above and below the surgical wound. The parameters of TENS therapy will be used for the GT group and in the combination therapy in L GT. The patient will be positioned in the supine position in a neutral position.

<u>LBI Intervention Protocol:</u>

The intervention protocol will use the study by Araújo et al (2019)7 as a reference. The LLLT will have a wavelength of 660nm (red) in continuous mode and will be performed in two sessions with an interval of 20 to 24 hours. The parameters of Low-Intensity Laser therapy are described in Table 01.

The technique will be punctual, without contact, and applied perpendicular to the skin at the line of the cesarean incision7. The number of stitches to be applied will depend on the extent of the surgical wound, respecting the distance of 1 cm between the application stitches (Figure 06). To perform the application with the LBI, the pen of the device must be sanitized with 70% alcohol, in which this procedure will be repeated for the intervention in each volunteer. Asepsis with 70% alcohol will be performed before and after the intervention in the region of the surgical wound. The patient's bed must be exclusively present by the therapist and the patient, who will use specific protective glasses of the device. The patient will be positioned in the supine position in a neutral position.

For the application of the LBI, it will be carried out in combination with the TENS, being applied in the GTL group

Table 01: Parameters of the LBI protocol.

**Parameters**	**Values**
Wavelength	660nm (vermelho)

Emission Mode Power	Continuous mode 100Mw
Dose	2J/cm2
Energy	0.12J
Time of application per point	4 seconds

Source: 7

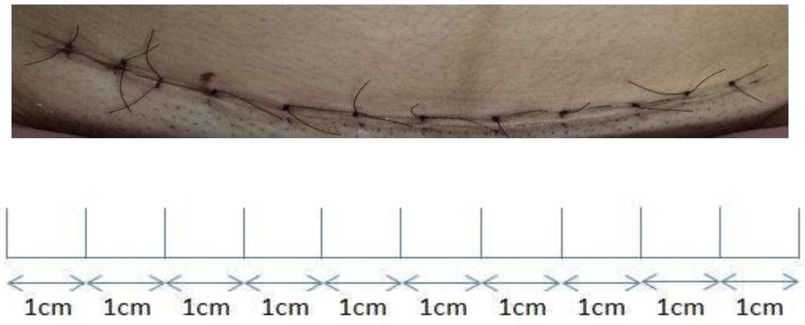

Figura 06: Irradiação do LBI com distance of 1cm between the application points.

5.5.1 Dias e horários das coletas

Collections will be carried out between 7 am and 10 am, on three consecutive days (Wednesdays, Thursdays and Fridays). All collections will be carried out in the postpartum joint accommodations of the Divino Amor Maternity (Parnamirim/RN).

5.6 Statistical analysis

For statistical analysis, the data will be analyzed using the SPSS 20.0 (*Statistical Package for the Social Sciences)* software for Windows 23.0, assigning a significance level < 5%.

Initially, a descriptive analysis of the study variables will be carried out through measures of central tendency and dispersion in order to characterize the sample, which will be displayed in the form of a table with their respective means and standard deviation according to the Kolmogorov-Sminorv normality test. For comparisons of means between groups (GC, GCP, GT and GTL) the ANOVA test will be used, if probable differences are verified, Tukey's post hoc test will be applied.

5.7 Study flowchart

The stages of the research are shown in the flowchart below, according to the methodological procedure proposed in the study.

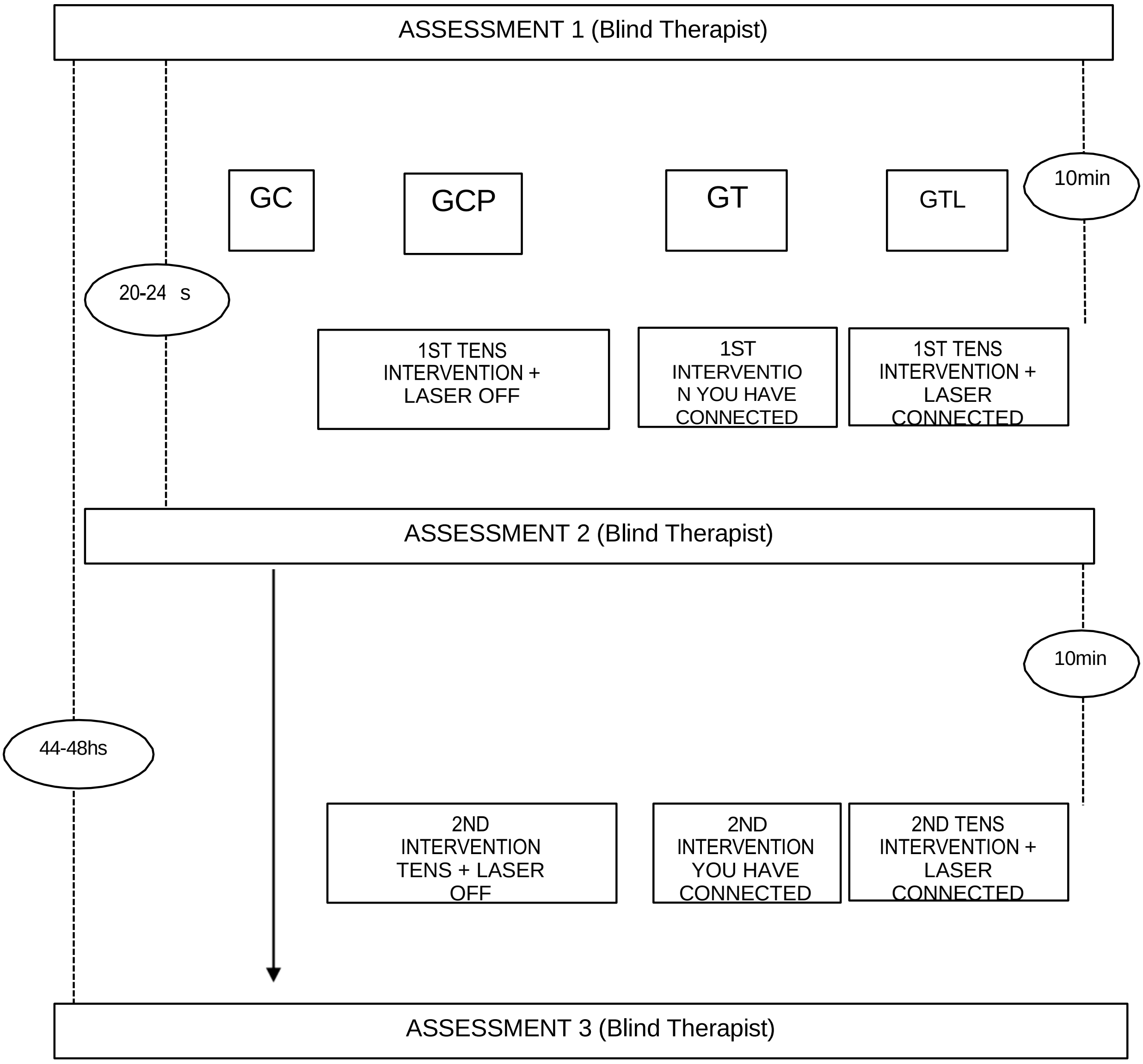

6. SCHEDULE

	2021 ii semester	2022 i semester	2022 ii semester	2023 i semester	2023 ii semester
Update Bibliographical	X	X	X	X	
Submission of the Project to the committee of ethics and research	X				
Data Collection		X	X		
Data Analysis				X	X
Qualification					X
Defense					X
Paper Submission					X

7. EXPECTED RESULTS AND PERSPECTIVES FOR THE ADVANCEMENT OF KNOWLEDGE

With the study, it is expected to verify better functional performance after the application of two sessions of combined therapy of TENS with LLLT for pain in the region of the cesarean incision. The research will allow physiotherapists and the academic physiotherapy community to use the combined therapy of TENS with LLLT as physiotherapeutic resources in patients undergoing cesarean section who are in the immediate puerperium, as well as will favor scientific evidence for the applicability of combined therapy in the relief of painful symptoms with improvement in functional performance in new studies.

8. RISKS AND BENEFITS

This research has minimal risks because it is a non-invasive evaluation and treatment. This research has minimal risks because it is a non-invasive evaluation and treatment. The volunteer may present pain z3 on the Numerical Pain Scale and thus be afraid during the evaluation with an algometer, and the researcher reinforces that the algometry exam aims to evaluate the perception of the discomfort of pressure and not to increase pain. Another possible risk will be infection at the site of the cesarean section, but this possibility is minimal since asepsis will be performed before and after the application with low-level laser and transcutaneous electrical nerve stimulation at the site of the electrodes. There may be possibilities of falling when performing the 2-minute Walk Test, but during this procedure the therapist will be close for greater safety during this evaluation. You may also have some questions about the intervention, the use of the transcutaneous electrical nerve stimulation resource, which will be used if the volunteer is part of the intervention group, consists of a technique with transmission of electric current and for this reason there may be risks of shocks but the device will be calibrated to perform the procedure, and the device will be prepared and tested for operation before the intervention and during use it will be monitored by the therapist Be careful not to have contact with liquids near the connected device. The TENS resource may also allow some discomfort in the skin by generating tingling sensations caused by the stimulus of the electric current, but the intensity will be according to the limit allowed by the volunteers, being started with zero intensity and increasing gradatively. Another risk that may arise from the applicability of TENS is that you may have skin irritation at the site of electrode placement, for this situation monitoring will be done during the session, in case of irritation the therapy will be suspended and the area at the electrode site will be cleaned. You may also have some questions about the intervention. There may be possibilities of falling when performing the 2-minute Walk Test, but during this procedure, the therapist will be close to the patient for greater sequence during this evaluation. Contamination by the coronavirus may exist, but all safety protocols to avoid contamination will be used, such as hand hygiene, use of

gloves, use of masks and the therapists responsible for the assessment and intervention will be vaccinated.

You may also have some questions about the intervention. The researcher will reinforce the benefits of the research by explaining all the stages of the research, emphasizing that it is expected that the application of low-intensity laser with TENS will reduce postpartum cesarean section pain, improving performance in performing activities that require movement in the first days after delivery and the importance of research for quality of life and health care.

9. BUDGET

ITEM	**QUANTITY**	**UNIT PRICE (R$)**	**TOTAL (R$)**
70% alcohol	3 liters	5,00	15,00
Key money	3 boxes	15,00	45,00
Paper Sheet	3 Rolls	15,00	45,00
Prints	250	0.10 (sheet)	45,00
Envelopes	300	0,40	120,00
Digital Algometer*	1	Available	Available
Laser de Baixa Intensity*	1	Available	Available
TENS”	1	Available	Available
Digital stopwatch*	1	Available	Available
Adhesive tape	3 units	7,00	21,00
		**Total**	**291,00**

The marked items (*) are already available from the researchers, and no cost is required for them.

10. REFERENCES

1 KASAPOĞLU I, KASAPOĞLU AKSOY M, ÇETINKAYA DEMIR B, ALTAN L. The efficacy of transcutaneous electrical nerve stimulation therapy in pain control after cesarean section delivery associated with uterine contractions and abdominal incision. Turk J Phys Med Rehab 2020; 66(2):169-175.

2 LAVAND'HOMME P. Postoperative cesarean pain: real but is it preventable? Curr Opin Anaesthesiol. 2018;31:262-267.

3 BORGES N C, PEREIRA L V, DE MOURA L A, SILVA T C, PEDROSO C F. Predictors for Moderate to Severe Acute Postoperative Pain after Cesarean Section. Pain Research and Management. Pain Res Manag. 2016; 2016:5783817.

4 SOUSA L, PITANGUI A C R, GOMES F A, NAKANO A M S, FERREIRA C H J. Measurement and characteristics of post-cesarean section pain and the relationship to limitation of physical activities. Acta Paul Enferm 2009;22:741-747.

5 PEREIRA T R C, SOUZA F G, BELEZA A C S. Implications of pain in functional activities in immediate postpartum period according to the mode of delivery and parity: an observational. Brazilian Journal of Physical Therapy 2017; 21(1):37-43.

6 CRESSWELL B M C et al. Pregnancy and Childbirth. Measurement of maternal functioning during pregnancy and postpartum: findings from the cross-sectional WHO pilot study in Jamaica, Kenya, and Malawi. (2020) 20:518.

7 ARAÚJO A M P H, SENA K R R, FILHO E M S, PEGADO R, MICUSSI M T A B
C. Low-level laser therapy improves pain in postcesarean section: a randomized clinical trial. Lasers in Medical Science, 2019.

8 POURSALEHAN S, NESIOONPOUR S, AKHONDZADEH R, MOKMELI S. The Effect of Low-Level Laser on Postoperative Pain After Elective Cesarean Section. Anesth Pain Med. 2018; 8(6):e84195

9 KAYMAN-KOSE S, ARIOZ D T, TOKTAS H, KOKEN G, KANAT-PECTAS M, KOSE M, YILMAZER M. Transcutaneous electrical nerve stimulation (TENS) for pain control after vaginal delivery and cesarean section. J Matern Fetal Neonatal Medicine. 2014:1—4.

10 FABRE HSC et al. Anti-inflammatory and analgesic effects of low-level laser therapy on the postoperative healing process J Phys. Ther. Sci. 2015; 27(6)

11 PENG W W, TANG Z Y, ZHANG F R, LI H, KONG Y Z, IANNETTI GD, HU L.Neurobiological mechanisms of TENS-induced analgesia. Neurolmage 195 (2019) 396—408.

12 ELBOIM-GABYZON M, NAJJAR S A, SHTARKER H. Effects of transcutaneous electrical nerve stimulation (TENS) on acute postoperative pain intensity and mobility after hip fracture: A doubleblinded, randomized trial. Clinical Interventions in Aging 2019:14 1841—1850.

13 MIOT H A. Sample size in clinical and experimental studies. J Vasc Bras. 2011;10:275-278.

14 DOMINGUES L, CRUZ E. Cultural Adaptation and Contribution to the Validation of the Patient Global Impression of Change Scale. Ifisionline 2011; 2(1).

15 BORGES J B C, FERRERA D L M P, CARVALHO S M R, MARTINS A S, ANDRADE R R, SILVA M A M. Evaluation of pain intensity and functionality in the recent postoperative period of cardiac surgery. Braz J Cardiovasc Surg 2006; 21(4): 393-402.

16 SELMAN J P R, CAMARGO A A, SANTOS J, LANZA F C, CORSO S D. Reference Equation for the 2-Minute Walk Test in Adults and the Elderly. Respiratory care. 2014: 4: 57.

17 BOHANNON R W, WANG Y, GERSHON R C, Reliability, validity and minimal detectable change of 2-min walk test and 10-m walk test in frail older adults receiving day care and residential care. Archives of Physical Medicine and Rehabilitation 2015; 96:472-7

18 MATSUDO S, ARAÚJO T, MARSUDO V, ANDRADE D, ANDRADE E, OLIVEIRA L C, BRAGGION G. International physical activity questionnaire (IPAQ): a validity and reproducibility study in Brazil. Rev. bras. Act. fis. health; 6(2): 05-18, 2001.

APPENDIX

APPENDIX A - INFORMED CONSENT FORM (ICF)

FEDERAL UNIVERSITY OF RIO GRANDE DO NORTE HEALTH SCIENCES CENTER DEPARTMENT OF PHYSICAL THERAPY

INFORMED CONSENT FORM (ICF)

Clarification

This is an invitation for you to participate in the research: **"INFLUENCE ON THE FUNCTIONAL PERFORMANCE OF COMBINED LASER THERAPY AND TENS IN THE**
CESAREAN SECTION INCISION WITH PAIN: randomized clinical trial" whose principal investigator is Prof. Dr. Patrícia Angélica de Miranda Silva Nogueira.

This research aims to evaluate the effect of the treatment of transcutaneous electrical nerve stimulation combined with low-level laser for pain relief at the site of the surgical cesarean section and consequently improve the performance in performing activities that require mobility during hospitalization.

The study will consist of four groups of volunteers. In two groups, two sessions will be done using TENS and laser, one group with the devices turned off and the other with the devices on. A third group will be submitted only to TENS. There will be a research group that will not apply any treatment resources.

If you decide to participate, you can be part of one of the study groups and your participation in the group will be done through a lottery. During your stay in the hospital, you will be evaluated three times: first evaluation between 8-12 hours after delivery, second evaluation between 20-24 hours after delivery, and third evaluation between 44-48 hours after delivery.

Participant/Legal Guardian Rubric:	Researcher's Rubric:

During your stay in the hospital, you will be evaluated three times: first evaluation between 8-12 hours after delivery, second evaluation between 20-24 hours after delivery, and third evaluation between 44-48 hours after delivery.

The evaluations will consider: sociodemographic data (name, address, age, educational level, marital status, associated diseases, coronavirus infection); obstetric data (gestational age, parity, date of delivery, postpartum time); clinical data (information on pain such as improvement or worsening after combined therapy of TENS with laser, functional performance to perform movements).

To assess pain, an exam called algometry will be carried out with a device that has a rubber tip. During this exam you will say "started" when you feel pain due to the pressure made by the tip of the device, but the pressure will continue until you say "stop". In addition to this examination, pain will be assessed using a Visual Numerical Scale composed of numbers (0 to 10), where 0 (Zero) there is no pain and 10 (ten) maximum pain.

Improvement or worsening after combined therapy of TENS with laser will also be assessed; and, evaluation of functional independence with the use of a 2-minute Scale and Walk Test that will be according to their limit.

If you are part of the groups that will use laser, this device will be applied in the region above the surgical cut. This device has a pen that will be sanitized with 70% alcohol and will not be made direct contact with the skin. It will be necessary for you and the researcher to wear your own protective glasses to use the device. Your bed should have the curtains closed, with only you and the therapist present at the time of laser application.

For the evaluation, the time will be an average of 30 minutes and for the therapeutic procedure the time will be an average of 40 minutes.

As this research will have the possibility of video recording and photographic recording, consent will be requested through a form from all volunteers. I inform you that the researcher will ensure that the research is carried out in an appropriate and reserved environment to ensure the participant's privacy.

During the research, possible discomfort and possible risks may occur because it is an evaluation and non-invasive treatments.

Participant/Legal Guardian Rubric:	Researcher's Rubric:

You may have pain a3 on the Numerical Pain Scale and thus be afraid during the algometer evaluation, but the algometry exam aims to evaluate the perception of the discomfort of the pressure and not to increase the pain. Another possible risk will be infection at the site of the cesarean section, but this possibility is minimal since asepsis will be performed before and after the application with low-level laser and transcutaneous electrical nerve stimulation at the site of the electrodes. There may be possibilities of falling when performing the 2-minute Walk Test, but during this procedure the therapist will be close for greater safety during this evaluation. You may also have some questions about the intervention, the use of the transcutaneous electrical nerve stimulation resource, which will be used if you are part of the intervention group, consists of a technique with transmission of electric current and for this reason there may be risks of shocks but the device will be calibrated to perform the procedure, and the device will be prepared and tested for operation before the intervention and During use, the therapist will be monitored carefully so as not to have contact with liquids near the connected device. TENS may also allow some discomfort on the skin by generating tingling sensations caused by the stimulation of the electric current, but the intensity will be according to the limit allowed by you, being started with zero intensity and gradually increasing, you may also have skin irritation at the place of placement of the electrodes, for this situation monitoring will be done during the session, In case of irritation, therapy will be suspended and the area at the electrode site will be cleaned. Coronavirus contamination may exist, but all safety protocols

gloves, use of masks and the therapists responsible for the assessment and intervention will be vaccinated.

pto avoid contamination will be used as a higienizaç of the hands. Usage of

As a benefit of the research, you will be able to improve your performance to perform activities that require movement in the first days after childbirth. If you are in the group that did not undergo the intervention, you will be contributing to science, bringing answers to possible new therapies.

Participant/Legal Guardian Rubric:	Researcher's Rubric:

In case of complications or damage to health that you may have related to the research, it is up to the principal investigator to guarantee the right to full and free care, which will be provided with physiotherapeutic treatment and medical referrals.

Throughout the research period, you can ask your questions by calling the principal investigator Dr. Patrícia Angélica de Miranda Silva Nogueira, by contact: (84) 99173-9579.

You have the right to refuse to participate or withdraw your consent, at any stage of the survey, without any prejudice to you.

The data you will provide us with will be confidential and will only be disclosed in congresses or scientific publications, always anonymously, and there will be no disclosure of any data that can identify you. This data will be kept by the researcher responsible for this research in a safe place and for a period of 5 years.

Some expenses for your participation in this research, they will be assumed by the researcher and reimbursed to you.

If you suffer any damage arising from this research, whether immediate or delayed, foreseen or unforeseen, you will be indemnified.

If you have any questions about the ethics of this research, you should call the Research Ethics Committee UFRN - Lagoa Nova Campus Central (CEP Central/UFRN) — an institution that evaluates the ethics of research before it begins and provides protection to its participants — from the Federal University of Rio Grande do Norte, at (84) 3215-3135 or (84) 9.9193-6266, Email cepufrn@reitoria.ufrn.br. You can still go in person to the CEP headquarters, from Monday to Friday, from 8:00 am to 12:00 pm and from 2:00 pm to 6:00 pm, at Rua das Artes, sin. UFRN Central Campus. Lagoa Nova. Natal/RN. Zip Code: 59075-000.

This document was printed in two copies. One will stay with you and the other with the principal investigator Dr. Patrícia Angélica de Miranda Silva Nogueira.

Participant/Legal Guardian Rubric:	Researcher's Rubric:

Informed Consent

After having been clarified about the objectives, importance and how the data will be collected in this research, in addition to knowing the risks, discomforts and benefits that it will bring to me and having been aware of all my rights, I agree to participate in the research **"INFLUENCE ON THE FUNCTIONAL PERFORMANCE OF COMBINED LASER THERAPY AND TENS IN THE INCISION**

CESAREAN SECTION WITH PAIN: randomized clinical trial" and I authorize the disclosure of the information provided by me in congresses and/or scientific publications as long as no data can identify me.

____

Survey participant signature

Participant's fingerprint

Statement by the Principal Investigator

As the researcher responsible for the study **"INFLUENCE ON THE FUNCTIONAL PERFORMANCE OF COMBINED LASER AND TENS THERAPY IN THE**

CESAREAN SECTION INCISION WITH PAIN: randomized clinical trial", I declare that I assume full responsibility for faithfully complying with the methodological procedures and rights that were clarified and assured to the participant of this study, as well as maintaining secrecy and confidentiality about the identity of the same.

I also declare that I am aware that in non-compliance with the commitment assumed herein, I will violate the norms and guidelines proposed by Resolution 466/12 of the National Health Council – CNS, which regulates research involving human beings.

Christmas _/____/______

____

Dr. Patrícia Angélica de Miranda Silva Nogueira

APPENDIX B - EVALUATION SHEET

FEDERAL UNIVERSITY OF RIO GRANDE DO NORTE HEALTH SCIENCES CENTER DEPARTMENT OF PHYSICAL THERAPY

Volunteer code:__________________ Date of collection: __/____/_____

- Identification

Name:____Age:____________________
Telephone:___________________________Time of delivery:_________________
Neighborhood:________________City/Municipality:__________________________

- Gestational age: _________Parity: _________Labor: ( )Yes( )No
- Associated Diseases: ____
- COVID-19:

Had COVID-19 ( ) Yes ( ) No How many times? ____
Were you infected before or during pregnancy?____
In case of COVID-19 infection during pregnancy, what was the trimester of gestation? ( ) 1st Quarter ( ) 2nd Quarter ( ) 3rd Quarter

- Maid: ( ) Yes ( ) No Smoker: ( ) Yes ( )No Partner: ( ) Yes ( )No
- Educational Level: ________________________ Years of study:_____________

Literate	YES	NO
Primary	COMPLETE	INCOMPLETE
Fundamental	COMPLETE	INCOMPLETE
Medium	COMPLETE	INCOMPLETE
Superior	COMPLETE	INCOMPLETE

1. Digital algometer: _______________
2. 2-minute Walk Test (2MWT):___________________Dor (1 minute TC2M):______
3. Functional Independence Measure:

Categories	AV1	AV2	AV3
PERSONAL CARE			
Self Care			
Feeding			
Bathing			
Dressing upper torso			
Dressing lower torso			
Intimate hygiene			
MOBILITY			
Bed/Chair/Wheelchair			
Bathroom			
Shower/bath bath			
LOCOMOTION			
Floor/Wheelchair			
Stairs			
TOTAL			

ATTACHMENT

APPENDIX A - NUMERICAL PAIN SCALE

0	1	2	3	4	5	6	7	8	9	10
No Pain										Maximu m Pain

VERBAL COMMAND:

0 = no pain

1-3 = Brand's dor

4-6 = moderate

pain 7-10 = severe

pain

APPENDIX B - GLOBAL PERCEPTION OF CHANGE SCALE (PGIC version Portuguese)

Scale of Perception*s^o Global Change (PGIC English version) Name:

____Data: ___________

Main complaint: __________________________________

Since the beginning of the treatment at this institution, how do you describe the change (if any) In LIMITATIONS OF ACTIVITIES SYMPTOMS EMOTIONS And QUALITY OF LIFE in your overall in relation to your pain (select ONE option):

No changes (or the condition has worsened)	0	1
Almost the same, without any visible change		2
Slightly better, but no significant changes		3
With some improvements, but the change didn't make any real difference	0	4
Moderately better, with slight but significant change	9	5
Better, and with improvements that have made a real and useful difference	0	6
Much better, and with a considerable improvement that made all the difference		7

Adapted and Validated by: Domingues, L. & Cruz, E. (2011)
Email: lucia.domingues@ess.ips.pt
Copyright 2004, Hurst, H. & Bolton, J.

ANNEX C — International Physical Activity Questionnaire (IPAQ short version)

International Physical Activity Questionnaire (IPAQ short version)

Name. ____________
Date: _ Age : Sex: F) M ("›
You traoaiha of 'o ma Paid: ( ) Yes ( ) No
Ouant3s 'hours you Works by day: ___________
How many Years Complete you °stuóou: _
In general health care form:
(ExcelSit i) Very much ho« ( ) Good (, Regular ( ) Rulrn

We are interested in knowing that people are aware of their daily lives . This project is part of an ongoing study in different countries around the world. 7A: The people who are asked will help us to understand that we are in relation to people and other parents. The questions are related to the time you spend doing physical activity in a NORMAL, USUAL or USUAL week.The questions \ac'uem the activities you do at work, to go it a 'ugar a of !rc, nor leisure. for sport. by exercise or as part of your activities at home or in the garden. Its resposlks S3o MUITO \mpsrtanIes, Por fuvor resçonds oada questão mesmo que co.nsidere oue nâc é ntivc. Thank you for a partnership !

PcYra rr,0ponder the questions \embre that:
° at VIGOROUS physical vitJÂdes are those that are marked by a great physical effort and that make wait very long
• activities ^.isic MODERATE Are aquo!ns that require some physical effort and that they do rcspi• ra A LITTLE stronger than normal

To answer the questions, think only of the activities that you react to oor oor less than 10 minutes çA? nuAp de corda vez:

ta. In quarltofi Úias de umtt sorY\aiFIiT fiDFMüI, VOCÕ FOüliza a"i'7‹ VIGOROUS ITUDES for q_eIo less than 1U minutes ron"i"ni n.e, as by ux0mp.u run, *azer aerobic gyrus, play football. Pedal fast on the bike. Basketball workers do heavy work in the home, yard or garden. carry watering weights or quaIo.uer un›viüa04 that 'aba you sweat A LOT or increase k\UJTO your response or heart beats .

Days Lor WEEK ( ) N0nf\um

1b. In days when you do these vigorous activities for p.eq mene The minute timing, how much time on the iotai do you spend this activity:and activities por_cIig?
Carr. . _ _ Minutes:

2nd. In rjuztnto * days of a ‹ s‹ mana norma.. YOU LB3IÍZn activities MODERATED by ! menns 10 m.nu them continuous. as per 4xrmplo ¿edalar Had nn bike swim. dance. do light aerobic gymnastics. Playing volleyball reereaii\ o. Ear watering uses iGv"0.0, t42E"r domestic services! COS in the ass. in the yard or in the garden as sweeping. vacuum. take care of the garden. or any activity that makes you sweat .eve or moderately aumanteic your breath or batim0ntub of curacao {POfl PLEASE DO NOT TNCLMA WALK)

Days by WEEK ( ) None

2b. On err› days That you do these activities modified by powder. Ic minus 1d m!nutos contar.uos how long ns total you game making these ajiviJades gozclJa?

hours' _______ Minutos: _

3s In quantos.dias.de a normal week v 6 walks even:at least J0 minutes oont/nuos.in ca6a.or at work, as a transport lorma to go:from.one place to" another, even:leisure, for.pleasure or as a way of 6xâK ÍGIGIO†

diaB by SEMAFiA ( ) None

3"b. Us fingers on the what voó"6 Walks by D61O g'tBD0g 10 minutes Oniiúuos How long in total Gas Walking About for7 "

horas: ______Minutes: _____

4a. And the last pe@úntas in relation to the time you spend sitting at work, at home, at school or college. during: the top free. This includes the time you spend sitting in the office au studying; f82ando lx;4o from home; vis'rlando aMi8••. I9ndo a &0ntado ou de'itadô assisôndo telavisâü.

ÕuantD"top per day.you sit on one day of the week*

hours: ______Minutes: _____

4"b. Oúdnto te'npo Pain day you sit on the "weekend weekend †"

______ _____
